# Integrated multi‐omic analyses uncover the effects of aging on cell‐type regulation in glucose‐responsive tissues

**DOI:** 10.1111/acel.14199

**Published:** 2024-06-26

**Authors:** Peng Xu, Yimeng Kong, Nicholette D. Palmer, Maggie C. Y. Ng, Bin Zhang, Swapan K. Das

**Affiliations:** ^1^ Center of Clinical Laboratory Medicine, Zhongda Hospital, School of Medicine, Advanced Institute for Life and Health Southeast University Nanjing China; ^2^ Department of Genetics & Genomic Sciences, Mount Sinai Center for Transformative Disease Modeling, Icahn Genomics Institute Icahn School of Medicine at Mount Sinai New York New York USA; ^3^ Department of Pharmacological Sciences, Mount Sinai Center for Transformative Disease Modeling, Icahn Genomics Institute Icahn School of Medicine at Mount Sinai New York New York USA; ^4^ Department of Artificial Intelligence and Human Health, Mount Sinai Center for Transformative Disease Modeling, Icahn Genomics Institute Icahn School of Medicine at Mount Sinai New York New York USA; ^5^ Department of Biochemistry Wake Forest University School of Medicine Winston–Salem North Carolina USA; ^6^ Division of Genetic Medicine, Vanderbilt Genetics Institute Vanderbilt University Medical Center Nashville Tennessee USA; ^7^ Department of Internal Medicine, Section on Endocrinology and Metabolism Wake Forest University School of Medicine Winston‐Salem North Carolina USA

**Keywords:** adipose, aging, cell‐type, metabolite, multi‐omic, muscle, network

## Abstract

Aging significantly influences cellular activity and metabolism in glucose‐responsive tissues, yet a comprehensive evaluation of the impacts of aging and associated cell‐type responses has been lacking. This study integrates transcriptomic, methylomic, single‐cell RNA sequencing, and metabolomic data to investigate aging‐related regulations in adipose and muscle tissues. Through coexpression network analysis of the adipose tissue, we identified aging‐associated network modules specific to certain cell types, including adipocytes and immune cells. Aging upregulates the metabolic functions of lysosomes and downregulates the branched‐chain amino acids (BCAAs) degradation pathway. Additionally, aging‐associated changes in cell proportions, methylation profiles, and single‐cell expressions were observed in the adipose. In the muscle tissue, aging was found to repress the metabolic processes of glycolysis and oxidative phosphorylation, along with reduced gene activity of fast‐twitch type II muscle fibers. Metabolomic profiling linked aging‐related alterations in plasma metabolites to gene expression in glucose‐responsive tissues, particularly in tRNA modifications, BCAA metabolism, and sex hormone signaling. Together, our multi‐omic analyses provide a comprehensive understanding of the impacts of aging on glucose‐responsive tissues and identify potential plasma biomarkers for these effects.

## Graphical abstract

This study comprehensively evaluates the impacts of aging on glucose‐responsive tissues by integrating multi‐omic data sets from the AAGMEx and GTEx cohorts. We identified aging‐associated network modules, metabolic changes, and cell‐type responses in adipose and muscle tissues. Our multi‐omic approach offers a deep understanding of aging effects and suggests potential plasma biomarkers for glucose‐responsive tissues.

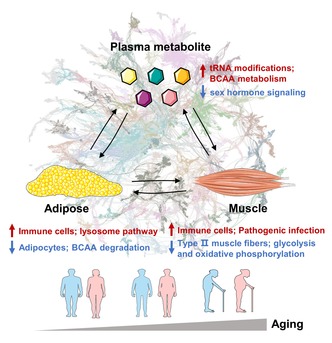

## 1 INTRODUCTION

Aging is a complex and multifactorial process characterized by the gradual accumulation of metabolic changes (Lopez‐Otin et al., 2023). One of the most prominent aging‐associated disorders is the impairment of nutrient‐sensing and physiological functions of glucose‐responsive tissues (Ou et al., 2022; Wilkinson et al., 2018). Adipose and muscle are the two major organs responsible for nutrient availability and energy metabolism: Adipose tissue plays a crucial role in storing lipids for energy homeostasis, whereas skeletal muscle accounts for approximately 80% of postprandial glucose disposal (DeFronzo & Tripathy, 2009; Kershaw & Flier, 2004). Understanding the gene‐regulatory networks involved in the rewiring of metabolism in these glucose‐responsive tissues during aging is critical to identify molecular regulators of the aging process and prioritize treatment strategies.

Adipose and muscle tissues are complex and heterogeneous, consisting of diverse cell types that are differentially impacted by the aging process. Aging is accompanied by a decrease in fat depot size and impairment in the differentiation of preadipocytes into adipocytes, along with elevated expression of antiadipogenic genes (Kirkland et al., 2002). In aged mice, visceral adipose tissue exhibits increased pro‐inflammatory macrophages, upregulated TNF‐α and IL‐6, and downregulated PPAR‐γ expressions (Wu et al., 2007). Aging also significantly impacts skeletal muscles, leading to sarcopenia, characterized by a decline in tissue mass and function (Sousa‐Victor et al., 2015). Although prior studies have highlighted the significance of cell‐type regulations in adipose and muscle tissues, a comprehensive understanding of aging‐related cell‐type specificity remains elusive.

Currently, single‐cell studies are widely employed to investigate cell type‐specific gene activities in glucose‐responsive tissues (De Micheli et al., 2020; Emont et al., 2022; Kedlian et al., 2024). However, these experiments are hindered by high costs and gene dropouts, limiting their application in large‐scale aging studies. To address these limitations, we previously developed the Multiscale Embedded Gene Co‐expression Network Analysis (MEGENA) approach. This method enables the direct inference of cell type‐specific high‐resolution network modules from bulk transcriptome data sets of human populations (Song & Zhang, 2015; Xu et al., 2022). Additionally, we have established the African American Genetics of Metabolism and Expression (AAGMEx) cohort, which includes comprehensive physiological and multi‐omic profiling of adipose and muscle tissues (Sajuthi et al., 2016; Sharma et al., 2016). By combining our MEGENA approach with the AAGMEx cohort, we aim to investigate cell type‐specific gene activities associated with aging in glucose‐responsive tissues.

Previous studies have identified certain plasma metabolites as potential indicators of the aging process (Chaleckis et al., 2016; Saito et al., 2016). However, the precise mechanistic connections between these biomarkers and the gene‐regulatory networks in glucose‐responsive tissues during aging remain incompletely understood. Leveraging the well‐curated AAGMEx cohort, we hypothesize that systematic omics data profiling can enable the detection of circulating metabolites as biomarkers reflecting inter‐tissue communication. Therefore, this study focuses on two principal objectives: (1) elucidating the aging‐related cell‐type networks within adipose and muscle tissues through an integrated examination of transcriptome, methylome, and single‐cell analyses and (2) identifying plasma metabolites as dependable biomarkers for intertissue communication during the aging process. By addressing these objectives, we aim to provide a comprehensive understanding of the influence of aging on cell‐type regulations and intercellular interactions within glucose‐responsive tissues.

## 2 RESULTS

### 2.1 Study design and analysis pipeline

We conducted a multi‐omic analysis on the AAGMEx cohort, which comprised 222 healthy participants (123 males and 99 females), spanning an age range of 18–61 years (Table S1). The AAGMEx cohort offers comprehensive participant data, including demographics such as age and sex, along with physiological metrics like body mass index (BMI) and glucometabolic measures (e.g., insulin sensitivity indices: S_I_ and Matsuda Index). The multi‐omic data set from the AAGMEx cohort encompasses transcriptomic data derived from microarrays, methylomic data obtained through reduced representation bisulfite sequencing (RRBS), and metabolomic data generated via untargeted liquid chromatography‐mass spectrometry (LC–MS). For the purpose of independent cohort validation, we utilized adipose and muscle tissue samples from the GTEx consortium, which includes 604 individuals aged between 21 and 70 years. The transcriptome of GTEx data set was acquired from postmortem tissues and sequenced using RNA sequencing (RNA‐seq) technology.

Our analysis workflow began with the application of MEGENA to adipose and muscle tissues, aiming to identify co‐expression networks and aging‐responsive modules (Figure S1). Subsequently, we integrated cell‐type markers from single‐cell studies to characterize aging‐associated cell type‐specific modules. To validate the transcriptome findings, we employed cell proportion deconvolution and conducted multi‐omic analyses on methylation and single‐cell data. Additionally, we performed metabolome profiling of blood plasma from the AAGMEx cohort to investigate aging‐related interactions between circulating metabolites and gene activities in glucose‐responsive tissues. Through pairwise module–module and metabolite–gene correlations across plasma, adipose, and muscle tissues, our analyses revealed tissue–tissue communications associated with the aging process.

### 2.2 Aging regulates cell type‐specific modules of adipose tissue

**FIGURE 1:**
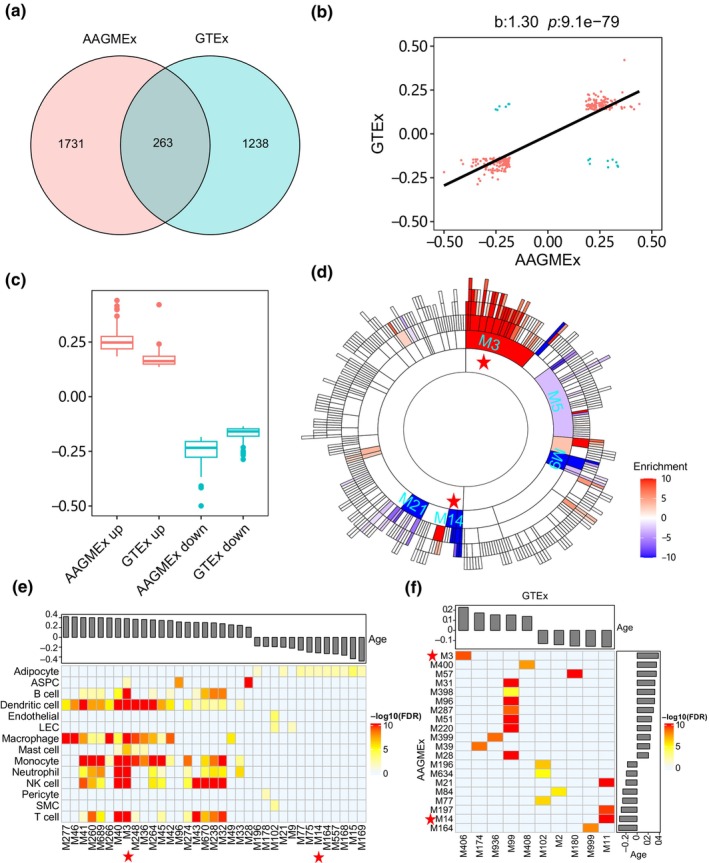
Aging‐associated genes and network modules in adipose tissue. (a) Venn diagram illustrating the overlap of aging‐related genes in the AAGMEx and GTEx cohorts. (b) Scatterplot comparing age correlation coefficients of aging‐related genes across AAGMEx and GTEx cohorts. The *x*‐ and *y*‐axes represent coefficients calculated using Spearman correlation in each cohort. (c) Distribution of Spearman coefficients for genes commonly associated with aging in both AAGMEx and GTEx cohorts. (d) Sunburst plot visualizing hierarchy of MEGENA network modules enriched for aging‐associated genes. The inner layers of the plot represent the parent modules of the outer layers. Color intensity indicates the enrichment significance, as measured by −log_10_(FDR). Positive values signify enrichments of aging‐upregulated genes, whereas negative values represent aging‐downregulated genes. Modules emphasized for downstream characterization are marked with red asterisks. (e) Heatmap depicting the cell‐type specificity of age‐related network modules in adipose tissue. The top bar plot shows the correlation coefficients between module eigengenes and age. ASPC, adipose stem and progenitor cells; LEC, lymphatic endothelial cell; SMC, smooth muscle cell. (f) Heatmap displaying the significance of Fisher's exact test for the preservation of top 20 AAGMEx modules in both cohorts. The accompanying bar plots indicate the correlation coefficients between module eigengenes and age.

We first explored the impacts of aging on gene expression within adipose tissue of the AAGMEx cohort. As linear regression analysis indicated a moderately significant correlation (*p* = 0.06) between age and BMI (Figure S2a), we normalized gene expression levels by regressing out the covariate effects of BMI and sex. Through Spearman correlation analysis on these normalized datasets, we identified 1994 genes associated with aging in adipose tissue, exhibiting a median absolute correlation coefficient of 0.23 and a False Discovery Rate (FDR) below 0.05 (Table S2). Subsequent multivariate regression analysis affirmed the aging association for 925 (89%) upregulated genes and 841 (88%) downregulated genes (FDR < 0.05) (Figure S2b). To corroborate these findings within the AAGMEx cohort, Spearman correlation analysis was applied to adipose tissue data from the GTEx consortium, identifying 1501 genes associated with aging, including 263 genes common to both cohorts (Figure 1a). Among these shared genes, 248 (94%) exhibited consistent aging correlation directions across both cohorts, while merely 15 (6%) displayed reversed directions, underscoring a general agreement in aging regulatory patterns (Figure 1b). Notably, the 248 consistently associated genes demonstrated stronger aging correlations in the AAGMEx cohort compared to the GTEx cohort, as evidenced by higher absolute Spearman correlation coefficients (Figure 1c). This indicates that despite its relatively smaller size, the AAGMEx cohort is particularly adept at detecting genes associated with aging.

Next, we constructed a MEGENA co‐expression network of the AAGMEx adipose tissue using covariates‐adjusted gene expressions. We identified 549 hierarchical modules, among which 75 modules were enriched for the aging‐upregulated genes and 52 modules were enriched for the aging‐downregulated genes (Fisher's exact test [FET], FDR < 0.05) (Table S3). The eigengenes (the first principal component of module expression) of top‐ranked modules, including M3 and M14, showed different trends of correlation with age (Figure 1d). By identifying cell‐type marker gene signatures from the single nucleus RNA‐sequencing (snRNA‐Seq) data set of adipose tissue (*n* = 57,599 nuclei) (Emont et al., 2022), we performed an enrichment test of network modules for the marker genes of each cell type in the adipose. We found that 34 aging‐associated modules (size >50 gene members) of the adipose were significantly enriched for the marker gene signatures of 12 different cell types (FET FDR < 0.05) (Figure 1e and Table S4). The major aging‐associated modules M3 and M14 were enriched for the maker gene signatures of immune cells and adipocytes, respectively. The modules from immune cells and adipose stem and progenitor cells (ASPCs) showed positive correlations with age, while the modules from the adipocytes negatively correlated with age.

To corroborate the network findings derived from the AAGMEx cohort, we performed MEGENA on the adipose tissue data from the GTEx consortium. The network preservation analysis showed that the principal cell type‐specific modules identified within the AAGMEx cohort are conserved within the GTEx dataset (Figure 1f). For example, the AAGMEx module M3, characterized by its enrichment in immune cells, aligns with the GTEx module M406, with both modules exhibiting a positive correlation with age. Similarly, the adipocyte‐specific module AAGMEx M14 and its counterpart in GTEx module M11 were negatively correlated with age. These results underscored the consistency of cell type‐specific aging responses across two independent cohorts.

### 2.3 Aging‐associated modules regulate metabolic pathways of adipose tissue

**FIGURE 2:**
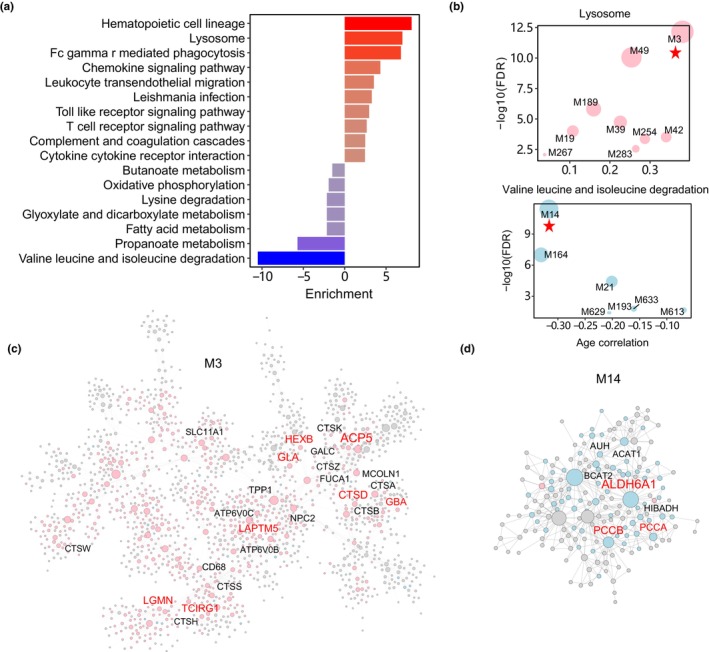
Metabolic pathways of aging‐associated network modules in adipose tissue. (a) KEGG pathway enrichment analysis of aging‐related genes in adipose tissue of the AAGMEx cohort. The top 10 most significantly enriched pathways are presented for genes exhibiting positive (indicated by red) and negative (indicated by blue) age correlations. (b) MEGENA module enrichment of lysosome and BCAA pathway genes. The *x*‐axis represents the correlation coefficient between module eigengene and age, whereas the *y*‐axis indicates the significance of enrichment. (c, d) Network visualizations of module M3 for the lysosome pathway (c) and M14 for BCAA degradation (d). Nodes represent genes, and links indicate co‐expression relationships. Node size correlates with network connectivity, and pink and blue colors represent positive and negative age correlations, respectively. The names of age‐associated genes from each pathway are labeled above the nodes, with red color indicating network hub genes.

To gain a deeper understanding of the impacts of aging, we annotated the aging‐associated genes and network modules in the AAGMEx adipose tissue using the KEGG pathway database. Our analysis revealed that genes upregulated by aging were enriched in hematopoietic cell development and lysosome pathways (Figure 2a). Conversely, genes downregulated by aging were enriched in the degradation of branched‐chain amino acids (BCAAs; including valine, leucine, and isoleucine), propanoate, and fatty acids. These metabolic pathways were also prominent in the aging‐associated cell type‐specific modules. Notably, the immune cell module M3 was positively correlated with age and stood out as the most enriched for the lysosome pathway (Figure 2b). Within M3, 24 genes from the lysosome pathway were upregulated by age, including key hub genes *HEXB* and *GBA* encoding lysosomal enzymes beta‐hexosaminidase and beta‐glucocerebrosidase, respectively (Figure 2c). On the other hand, the adipocyte module M14 was involved in BCAA degradation. In M14, seven genes involved in the BCAA degradation pathway were downregulated by age, such as *PCCA* and *PCCB* which encode the enzyme propionyl‐CoA carboxylase, a mitochondrial enzyme critical for BCAA catabolism (Figure 2d).

In previous studies, we have conducted extensive research on glucometabolic regulations within the AAGMEx cohort (Das & Das, 2024; Sharma et al., 2020; Xu et al., 2023). To further explore the relationship between aging and glucose response, we employed Spearman correlation analysis adjusted for age and sex to pinpoint the genes associated with BMI and insulin sensitivity (S_I_). Our findings revealed that in adipose tissue, 72% and 43% of aging‐associated genes were correlated with BMI and S_I_, respectively (Figure S2c). Additionally, through network module enrichment analysis, we discovered several aging‐associated modules enriched with BMI‐ and S_I_‐associated genes. Notably, modules M3 and M14, unique to immune cells and adipocytes, respectively, were enriched with BMI‐ and S_I_‐associated genes (Figure S2d). This finding indicates that these cell‐specific modules likely play dual roles in regulating both aging and glucose metabolism processes.

### 2.4 Aging regulates the proportion of different cell types and DNA methylation levels within adipose tissue

**FIGURE 3:**
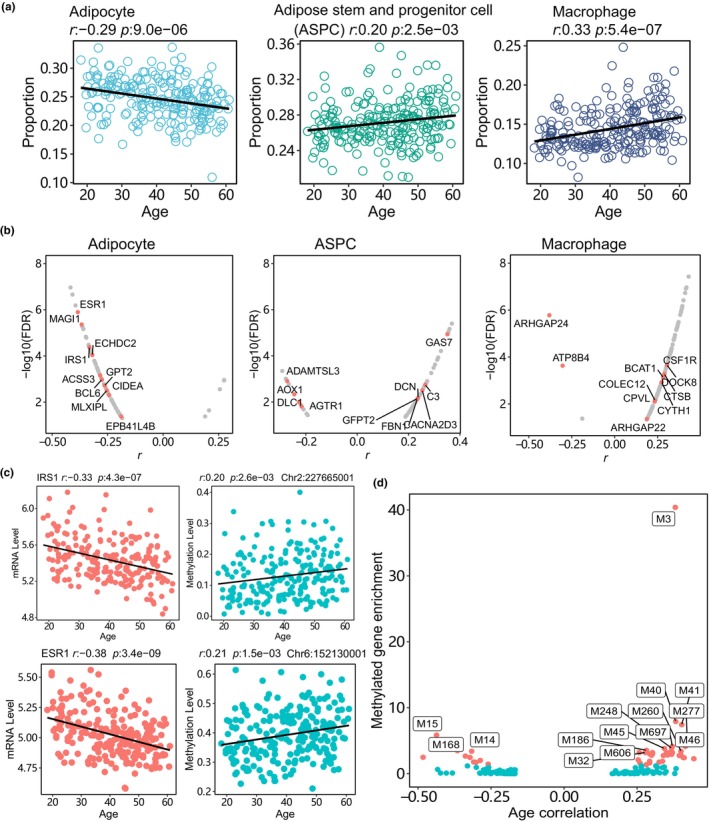
Aging‐associated cell‐type proportion and DNA methylation profile changes in adipose tissue. (a) Proportions of adipocytes, ASPC, and macrophages across different ages in the adipose tissue of the AAGMEx cohort. Cell‐type proportions were calculated using deconvolution analysis based on single‐nuclei transcriptome profiles. The black line in the point plot represents the slope of the linear regression model. (b) Volcano plots depicting gene expression and methylation alterations of aging‐associated marker genes in various adipose tissue cell types. Each dot represents an aging‐associated marker gene for a specific cell type. The *x*‐axis indicates the age correlation of gene expressions, whereas the *y*‐axis shows the correlation significance. Red dots indicate marker genes that also exhibit aging‐associated DNA methylation changes in the promoter region within a 1 kb window. (c) Aging‐associated expression and DNA methylation levels of representative adipose marker genes. Each panel displays the aging correlation of gene expressions on the left dot plot and the aging correlation of methylations in the promoter region on the right dot plot. (d) Enrichment of methylated aging‐associated genes in the adipose network modules. The correlation coefficients between module eigengenes and age are plotted on the *x*‐axis, whereas the enrichment significance, represented by −log_10_(FDR), is shown on the *y*‐axis.

To assess the impact of aging on cell‐type proportions, we employed bulk gene expression deconvolution to estimate the relative abundances of distinct cell types within the adipose tissue of the AAGMEx cohort. For this purpose, we utilized BisqueRNA, a method that relies on marker gene expression profiles derived from single‐cell experiment (Emont et al., 2022; Jew et al., 2020). Correlation analysis revealed that aging differentially affects the proportions of various cell types (Table S5). Notably, the proportion of adipocytes exhibited a negative correlation with age (Figure 3a), whereas the proportions of ASPCs and immune cells, including macrophages, positively correlated with age. These deconvolution results align with the observed changes in gene expression patterns of cell type‐specific modules, further highlighting the association between aging and alterations in cell‐type proportions.

Given the well‐established association between aging and epigenetic changes across various human tissue (Jones et al., 2015), we analyzed the gene methylation profiles with 1 kb tiling windows across the genome in adipose tissue of AAGMEx cohort. Using Spearman correlation analysis, we identified 2925 genes exhibiting aging‐related methylation patterns within a 1 kb window of their promoters (*p* < 0.05, Table S6). Among these, 428 genes demonstrated concurrent aging‐associated changes in both expression and DNA methylation levels, including 130 cell type‐specific marker genes. Specifically, 19, 20, and 35 marker genes were identified for adipocytes, ASPCs, and macrophages, respectively (Figure 3b and Table S7). *IRS1*, a gene crucial for insulin and IGF‐1‐related metabolic actions as well as adipocyte differentiation, displayed age‐associated downregulation in expression along with increased methylation levels (Figure 3c). Similarly, *ESR1*, encoding an estrogen receptor inversely associated with adiposity and positively associated with insulin sensitivity, also exhibited age‐related downregulation in expression along with elevated methylation. Beyond these genes, we also identified 2497 genes that solely exhibited aging‐associated methylation changes. For instance, *DIO3*, a gene regulating thyroid hormone metabolism, displayed elevated methylation levels with age (*r* = 0.47, *p* = 2e‐13), while its expression remained relatively unchanged. The genes that were associated with both aging and DNA methylation showed significant enrichment in 45 network modules, including the immune cell‐specific M3 module and the adipocyte‐specific M14 module (Figure 3d). It is worth noting that module M3 contained 149 genes (constituting 35% of the total genes), which were simultaneously influenced by aging and methylation. This underscores the intricate interplay between gene expression and methylation during the aging process in adipose tissue.

We conducted a detailed analysis of aging‐related gene expressions across different cell types using single‐nucleus RNA‐sequencing (snRNA‐seq) profiles from 13 individuals (mean age: 47 years, SD: ±15) (Emont et al., 2022). After adjusting for covariates such as body mass index (BMI) and sex, our pseudobulk analysis identified 45 genes significantly associated with aging (*p* < 0.05), which also exhibited consistent age‐related patterns in bulk RNA‐sequencing data from the AAGMEx adipose tissue cohort (Table S8). Notably, among these genes, *ANXA1*, a key regulator of immune responses and glucocorticoid‐mediated effect, demonstrated upregulation with aging in adipocytes (Figure S3a,b). *MAP3K8*, which controls the production of the pro‐inflammatory cytokine TNF‐alpha (TNF) during immune response, displayed a positive correlation with aging in macrophages (*r* = 0.56, *p* = 0.05). *ITK*, a crucial gene in immune cell module M3 within adipose tissue, regulates T cell development and differentiation and showed a positive correlation with age in T cells in our snRNA‐seq pseudobulk analysis (*r* = 0.65, *p* = 0.02). These findings further support the role of these aging‐associated genes in adipose tissue development across different cell types.

### 2.5 Aging regulates cell type‐specific network modules of muscle tissue

**FIGURE 4:**
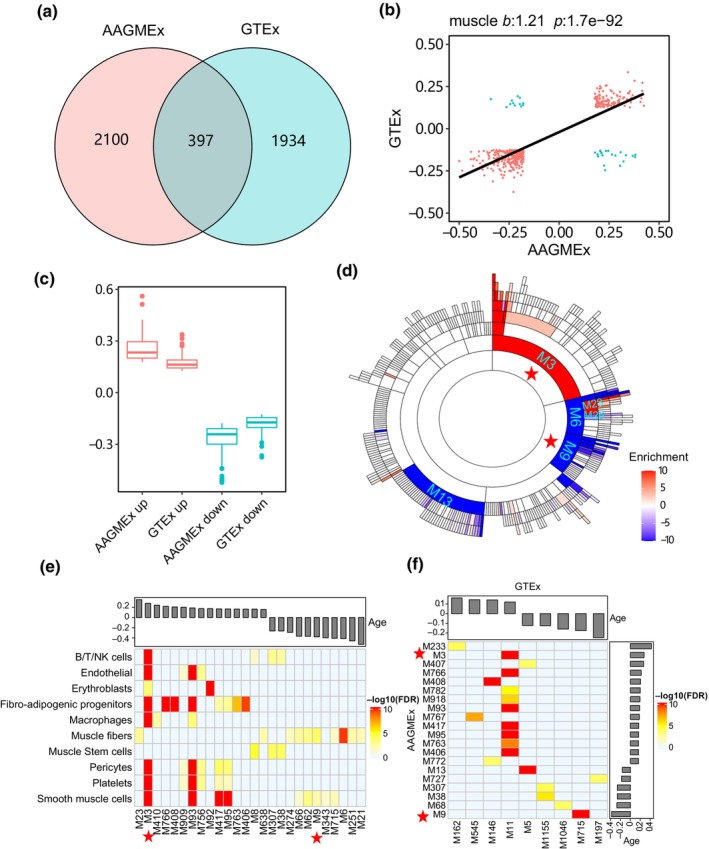
Aging‐associated genes and network modules in muscle tissue. (a) Venn diagram depicting aging‐associated genes in muscle tissue from the AAGMEx and GTEx cohorts. (b) Point plots exhibiting the concordance of age correlation coefficients for aging‐associated genes across both the AAGMEx and GTEx cohorts. The *x*‐ and *y*‐axes represent coefficients calculated using Spearman correlation in each cohort. (c) Distribution of Spearman coefficients for aging‐associated genes in both the AAGMEx and GTEx cohorts. (d) Sunburst plot visualizing hierarchy of MEGENA network modules enriched for aging‐associated genes. The inner layers of the plot represent the parent modules of the outer layers. Color intensity indicates the enrichment significance, as measured by −log_10_(FDR). Positive values signify enrichments of aging‐upregulated genes, whereas negative values represent aging‐downregulated genes. Red asterisks highlight modules for downstream analysis. (e) Cell‐type specificity of age‐associated network modules in muscle tissue. The heatmap illustrates the enrichment of cell‐type markers within the network modules. The bar plot at the top displays the correlation coefficients between module eigengenes and age. (f) Preservation of aging‐associated modules in the AAGMEx and GTEx cohorts. The heatmap shows the significance of Fisher's exact test for the top 20 preserved AAGMEx modules. The bar plots indicate the correlation coefficients between module eigengenes and age.

We further conducted a detailed investigation of the impact of aging on gene expression in muscle tissue from the AAGMEx cohort. After adjusting for BMI and sex covariates, we employed Spearman correlation analysis and identified 2497 genes significantly associated with aging (FDR < 0.05), which exhibited a median absolute correlation coefficient of 0.23 (Table S9). Using a multivariate regression model, we confirmed the association of 1101 (94%) aging‐upregulated genes and 1203 (91%) aging downregulated genes (Figure S4a). To validate our findings in an independent cohort, we performed Spearman correlation analysis on the GTEx data set. This analysis revealed 2331 aging‐associated genes in muscle tissue, including 397 genes commonly associated with aging in both cohorts (Figure 4a). Among the shared aging‐associated genes, 365 (92%) genes exhibited the same direction of aging correlation, whereas only 32 (8%) genes showed the opposite direction (Figure 4b). Consistent with our observations in adipose tissue, the 365 conserved genes demonstrated a stronger correlation with aging in the AAGMEx cohort compared with the GTEx cohort (Figure 4c).

Using the MEGENA pipeline, we identified 521 hierarchical modules from the co‐expression network in muscle tissue. Among these, 49 modules were enriched for aging‐upregulated genes, whereas 66 modules were enriched for aging‐downregulated genes (FET FDR < 0.05) (Table S10). The top‐ranked aging‐associated modules included M3, which positively correlated with aging, and M9, which was negatively regulated by aging (Figure 4d). To gain further insights, we analyzed single‐cell transcriptome profiles (*n* = 22,000 cells) of human muscle tissue and identified network modules specific to different cell types (De Micheli et al., 2020). The enrichment test revealed that 26 aging‐associated modules preferentially expressed marker genes of 10 cell types. Modules including M3 were enriched for marker genes from endothelial cells and immune cells, whereas modules including M9 were primarily derived from muscle fiber cells (Figure 4e and Table S11). Additionally, network preservation analysis demonstrated that major cell type‐specific modules in the AAGMEx cohort, such as M3 and M9, were also conserved in the GTEx cohort (Figure 4f).

We further explored the impact of BMI and insulin sensitivity (S_I_) on aging‐associated genes in the muscle tissue. We found that 10% and 6% of aging‐related genes were correlated with BMI and S_I_, respectively (Figure S4b). Several aging‐associated modules were enriched for the BMI‐ and S_I_‐linked genes. Notably, module M9, which was negatively correlated with age and unique to muscle fiber cells, was enriched for BMI‐ and S_I_‐associated genes (Figure S4c), suggesting its role in both aging process and glucose response.

### 2.6 Aging‐associated modules participate in metabolic regulation of muscle tissue

**FIGURE 5:**
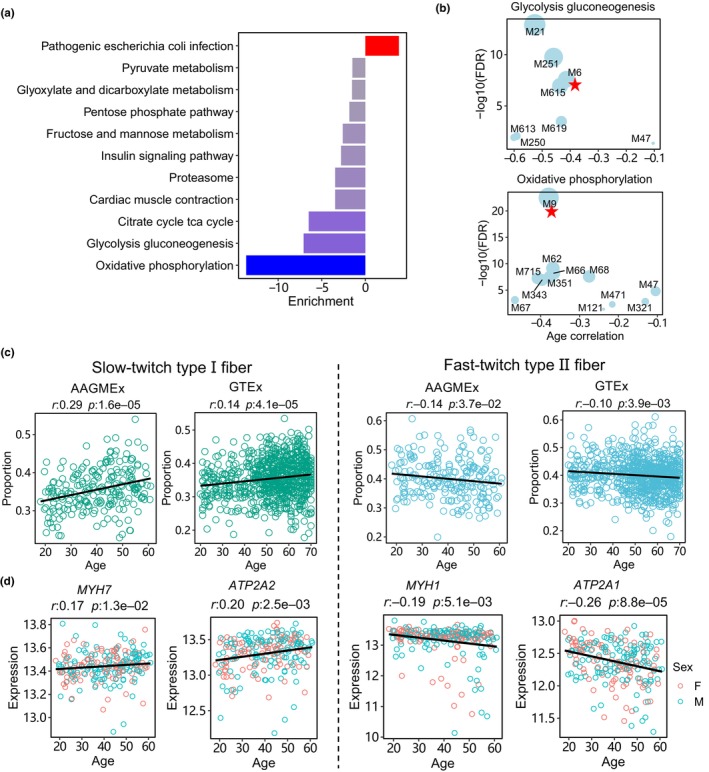
Aging‐associated metabolic pathways and muscle fiber types in muscle tissue. (a) KEGG pathway enrichment analysis of aging‐associated genes in muscle tissue of the AAGMEx cohort. The top 10 most significantly enriched pathways are presented for genes exhibiting positive (indicated by red) and negative (indicated by blue) age correlations. (b) MEGENA module enrichment for glycolysis and oxidative phosphorylation genes. The *x*‐axis represents the correlation coefficient between module eigengene and age, whereas the *y*‐axis indicates enrichment significance. (c) Proportions of slow and fast muscle fibers with age in the adipose tissue of the AAGMEx cohort. Cell‐type proportions were calculated using deconvolution analysis based on single‐nuclei transcriptome profiles. The black line in the point plot represents the slope of the linear regression model. (d) Dot plots depicting age correlations with marker gene expressions in different muscle fiber types. *MYH7* and *ATP2A2* for slow‐twitch type I fibers; *MYH1* and *ATP2A1* for fast‐twitch type II fibers. Red and blue colors represent female and male samples, respectively. The black line indicates the slope of the linear regression model.

By annotating the network modules using KEGG pathways, we discovered that the pathogenic infection pathway was enriched among the aging‐upregulated genes. GO database annotation further broadened our understanding of the gene functions of these upregulated genes, revealing categories such as “RNA splicing” and “actin filament organization” among the top 10 ranked GO processes. On the other hand, the aging‐downregulated genes impacted several crucial metabolic pathways in the KEGG database, including oxidative phosphorylation, glycolysis, and gluconeogenesis (Figure 5a). Among the aging‐associated modules involved in metabolism, M6 was enriched for glycolysis and gluconeogenesis. It comprised 961 genes, including 12 genes of the glycolysis and gluconeogenesis pathway downregulated by age (Figure 5b). For instance, *PFKM* encodes 6‐phosphofructokinase, which catalyzes the phosphorylation of fructose 6‐phosphate to fructose 1,6‐bisphosphate, representing the major rate‐limiting step of glycolysis (Schöneberg et al., 2013) (Figure S5a). Additionally, M9 was enriched for the oxidative phosphorylation pathway and contained 837 module genes. In M9, 32 genes of the oxidative phosphorylation pathway were downregulated by age, including hub genes *CYC1*, *COX5B*, and *UQCRH* (Figure S5b). These genes encode enzymes of the mitochondrial respiratory chain, crucial for oxidative phosphorylation functions.

### 2.7 Aging regulates proportion and gene expression of different muscle fiber types

Skeletal muscles encompass two distinct muscle fiber types: slow‐twitch (type I) and fast‐twitch (type II). Previous studies have demonstrated that aging leads to preferential loss and atrophy of glycolytic, fast‐twitch type II muscle fiber, indicating differential regulation of muscle fiber types by aging (Akasaki et al., 2014). To further investigate this, we conducted deconvolution analysis on muscle tissues from the AAGMEx and GTEx cohorts, utilizing snRNA‐Seq expression profiles (Jew et al., 2020; Perez et al., 2022). Our analysis revealed an increase in the proportion of slow‐twitch type I muscle fibers and a decrease in the proportion of fast‐twitch type II muscle fibers with age in both cohorts (Figure 5c). Consistent with this observation, we found that *MYH7* and *MYH1*, markers of slowt‐witch type I and fast‐twitch type II fibers, respectively (Schiaffino & Reggiani, 2011), correlated with age in opposite directions in the AAGMEx cohort (Figure 5d). This finding was corroborated by a parallel marker gene set from the sarcoendoplasmic reticulum calcium transport ATPase, including *ATP2A2* (marker of type I fiber) and *ATP2A1* (marker of type II fiber) (Periasamy & Kalyanasundaram, 2007). At the network level, the type I fiber marker genes (*MYH7* and *ATP2A2*) were co‐expressed in M23, which was enriched for aging‐upregulated genes (FET FDR = 1.2e‐24), whereas the type II fiber marker genes (*MYH1* and *ATP2A1*) were co‐expressed in M26, which was enriched for aging‐downregulated genes (FET FDR = 3.6e‐07) (Figure S5c). These results suggest distinct proportions and gene activities of muscle fiber types in response to the aging process.

### 2.8 Aging regulates metabolome network in blood plasma

**FIGURE 6:**
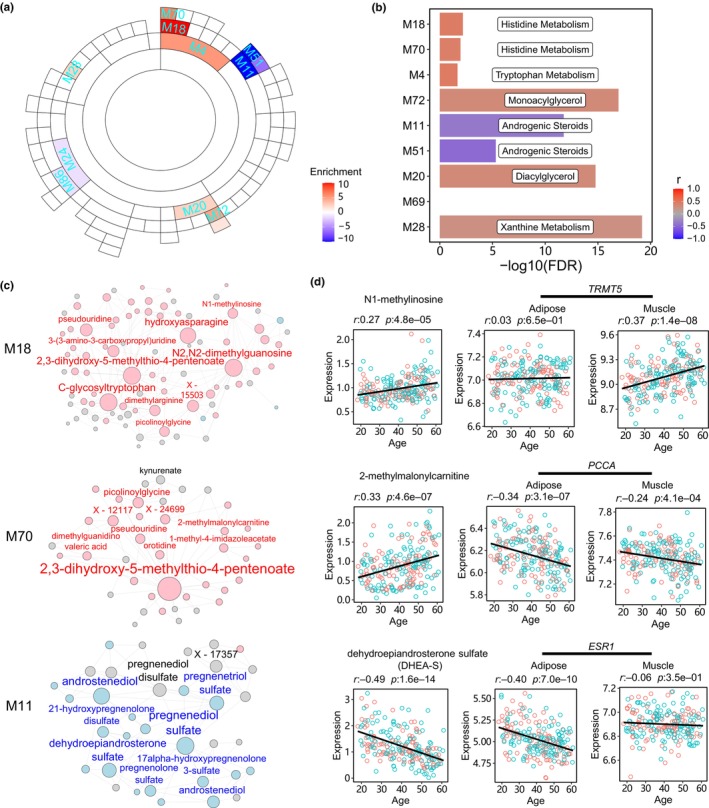
The network modules of plasma metabolites and their interactions with adipose and muscle genes. (a) Sunburst plot visualizing hierarchy of MEGENA network modules enriched for aging‐associated metabolites. The inner layers of the plot represent the parent modules of the outer layers. Color intensity indicates the enrichment significance, as measured by −log_10_(FDR). Positive values signify enrichments of aging‐upregulated genes, whereas negative values represent aging downregulated genes. (b) Functional annotations of network modules enriched for age‐associated metabolites. (c) Network plots of top‐ranked aging‐associated modules. Nodes represent metabolites, and lines indicate co‐regulation relationships. Hub metabolite names are labeled above the nodes. Node color (pink/blue) and metabolite names indicate positive/negative age correlations. (d) Examples of age‐associated metabolites and their biosynthesis or pathway genes in adipose and muscle tissues. Dot plots depict correlations between age and metabolite/gene expression levels. Red and blue colors represent female and male samples, respectively. The black line represents the slope of the linear regression model.

We conducted a thorough investigation to assess the impact of gene activities in adipose and muscle tissues on the levels of circulating metabolites during aging. Utilizing LC–MS, we profiled the metabolome of blood plasma from the AAGMEx cohort and successfully quantified 1124 metabolites in the plasma. After adjusting for BMI and sex, we identified 267 metabolites that were positively correlated with age and 61 metabolites that negatively correlated with age using the Spearman correlation test (FDR < 0.05). Subsequently, a multivariate linear regression model confirmed 221 (83%) aging‐upregulated metabolites and 43 (70%) aging‐downregulated metabolites (FDR < 0.05) (Table S12 and Figure S6). This observed difference might be attributed to the fact that the Spearman correlation excels at measuring the strength and direction of monotonic relationships between variables without assuming linearity. Furthermore, using MEGENA for metabolic network construction, we identified seven modules enriched for aging‐upregulated metabolites and five modules enriched for aging‐downregulated metabolites (Figure 6a and Table S13). Among these, the top‐ranked modules included hierarchical modules M4, M18, and M70, which positively correlated with age and were involved in amino acid metabolism pathways (Figure 6b). On the other hand, M11 emerged as a top aging‐downregulated module, playing a crucial role in androgenic steroid metabolism.

To identify the interactions between plasma components and glucose‐responsive tissues, we conducted module‐wise correlation analyses involving the aging‐associated modules derived from plasma metabolome data and the transcriptome data of adipose and muscle tissues. The Spearman correlation analysis unveiled significant associations between the top‐ranking aging‐associated modules found in plasma (e.g., M18, M70, M11) and the corresponding aging‐associated gene modules in adipose and muscle tissues (Figure S7a,b). Moreover, we also identified substantial correlations between the high‐ranking aging‐associated modules within adipose and muscle tissues themselves (Figure S7c). Significant correlations were observed between the adipose immune cell module M3 and the muscle fiber modules M23 and M26. Specifically, a positive correlation was found between adipose M3 and type I muscle fiber module M23 (*r* = 0.27, FDR = 0.004), while a negative correlation existed between adipose M3 and type II muscle fiber module M26 (*r* = −0.21, FDR = 0.04).

Given that adipose tissue is a crucial endocrine organ contributing to a pro‐inflammatory environment, and that adipose‐derived factors regulate mitochondrial function in skeletal muscle (Parra‐Peralbo et al., 2021; Seldin et al., 2018), the observed associations imply inter‐tissue communication influenced by the aging process within the human body.

### 2.9 Plasma metabolites reflect aging‐associated gene activities of glucose‐responsive tissues

To gain a deeper understanding of the roles of circulating biomarkers during the aging process, we conducted a thorough analysis of metabolite networks and aging‐related modules. We found that module M18 contained 89 metabolites and was positively correlated with age (*r* = 0.4, FDR = 1.5e‐10). Among the hub metabolites of M18, three compounds, namely N1‐methylinosine, N2,N2‐dimethylguanosine, and N6‐carbamoylthreonyladenosine, are involved in posttranscriptional chemical modifications of the tRNA maturation process (Figure 6c). The corresponding biosynthetic enzyme‐encoding genes of the three compounds, including *TRMT5*, *TRMT1*, and *YRDC* (de Crecy‐Lagard et al., 2019), also positively correlated with age in adipose or muscle tissues (Figure 6d and Figure S8). Correlation analysis further revealed that the plasma level of N1‐methylinosine positively correlated with the biosynthetic gene *TRMT5* in both adipose (*r* = 0.13, *p* = 0.05) and muscle (*r* = 0.19, *p* = 0.005) tissues.

Module M70, a sub‐module of M18, contained 39 metabolites. A key compound within M70 was 2‐methylmalonylcarnitine, a metabolite upregulated during aging and a byproduct of BCAA catabolism (Figure 6c,d). Consistent with these findings, the propionyl‐CoA carboxylase (encoded by *PCCA* and *PCC*) responsible for BCAA degradation was downregulated with aging in both adipose and muscle tissues. The plasma levels of 2‐methylmalonylcarnitine negatively correlated with *PCCA* (*r* = −0.18, *p* = 0.008) and *PCCB* (*r* = −0.20, *p* = 0.003) expression in adipose tissue. Furthermore, we found that the α‐subunit encoding gene of succinyl‐CoA ligase (*SUCLG1*) was downregulated with aging in both adipose and muscle tissues (Figure S8). This supports the hypothesis that the accumulation of 2‐methylmalonylcarnitine in plasma may also be attributed to the reduced function of succinyl‐CoA ligase, as previous report (Van Hove et al., 2010).

The primary aging‐downregulated module, M11, encompassed 34 metabolites and exhibited a negative correlation with age (*r* = −0.4, FDR = 1e‐06). The hub compounds of M11 primarily consisted of sex hormone intermediates, including dehydroepiandrosterone sulfate (DHEA‐S) (Figure 6c,d). These sex hormone intermediates were downregulated with aging in both males and females. Notably, the plasma level of DHEA‐S positively correlated with the estrogen receptor *ESR1* in adipose tissue (*r* = 0.24, *p* = 0.003) but not in muscle tissue (*r* = 0.005, *p* = 0.48). This suggests that estrogen signaling plays a crucial role in the deposition and metabolism of adipose tissue in both sexes (Cooke et al., 2017). Consistent with this, the expression of the estrogen receptor‐encoding gene *ESR1* decreased with age in adipose tissue, while the expression of the androgen receptor‐encoding gene *AR* remained relatively unchanged (Figure S8).

## 3 DISCUSSION

In this study, we conducted a comprehensive network analysis of adipose and muscle tissues to investigate aging‐associated regulations across different cell types and circulating metabolite biomarkers. Through network modeling, we revealed cell‐type‐specific modules during aging in adipose tissue, including reduced adipocyte activity and enhanced immune cell activity. By integrating multi‐omic data, we revealed that aging has cell‐type‐specific effects on gene expressions, methylations, and cell proportions in adipose tissue. In muscle tissue, aging downregulated metabolic functions in oxidative phosphorylation, glycolysis, and gluconeogenesis. It also repressed gene expressions in fast‐twitch type II muscle fibers while upregulating activities of slow‐twitch type I muscle fibers. Furthermore, we observed correlations between plasma metabolites and corresponding gene expression levels in glucose‐responsive tissues, indicating coordinated regulations of cross‐tissue metabolism during the aging process. Overall, our network analyses provided a comprehensive understanding of the cell‐type‐specific landscape involved in the aging process of adipose and muscle tissues.

We identified three representative plasma compounds that serve as potential biomarkers of the aging process: enhanced tRNA modifications, accumulation of BCAA byproducts, and reduced sex hormone molecules. tRNAs, which are transcribed by RNA polymerase III and involved in protein biosynthesis, exhibit increased levels with aging and potentially indicate mitochondrial dysfunction (Dluzen et al., 2018). Enhanced tRNA modifications might reflect aging‐related changes in mitochondrial activity and cellular metabolism. BCAAs include leucine, isoleucine, and valine that can activate mTORC1 signaling (Wolfson et al., 2016). Dietary BCAA restriction promotes metabolic health and lifespan extension in mice (Richardson et al., 2021). Consistent with this, we observed repressed BCAA degradation in adipose tissue and accumulation of BCAA byproducts in plasma during aging. Furthermore, we found that aging is associated with reduced sex hormone compounds and downregulated expression of *ESR1* in adipose tissue. Previous studies have shown that *ESR1* deletion leads to increased adiposity, fibrosis, insulin resistance, and glucose intolerance in both male and female mice (Heine et al., 2000). Therefore, the decreased plasma levels of sex hormone compounds may reflect the progressive age‐related decline in hormone production and action.

We also found that network modules of aging‐associated cell types (immune cells, adipocytes, and muscle fiber cells) were enriched for BMI‐associated genes, indicating that these cell types were regulated by obesity. The association of specific cell types in adipose and muscle tissues with obesity and diabetes was reported in recent studies. For example, deconvolution analysis of adipose tissues showed that the proportion of adipocytes and immune cells was correlated with BMI (Emont et al., 2022); single‐cell experiment identified fibro‐adipogenic progenitor cells associated with extracellular matrix remodeling in muscle tissues of diabetic patients (Farup et al., 2021); Population studies revealed that BCAA metabolism was associated with incidence of diabetes (Chai et al., 2022). Since aging‐associated cell types are crucial for energy metabolism, they may play dual roles in the physiological changes of aging and obesity. Targeting aging‐vulnerable cell types, such as lifestyle modifications through the exercise program and calorie restriction, may provide therapeutic clues to alleviate aging‐associated metabolic disorders (Pataky et al., 2021). Our study suggests that the aging process and a constellation of major chronic diseases, including obesity, diabetes, and cardiovascular diseases, likely share common metabolic, inflammatory, and epigenetic drivers. These conditions may be prevented potentially by modulating common targets with single interventions.

Although we conducted independent cohort validations to confirm aging‐associated regulations, the issue of noise and the absence of epigenomic and metabolomic data in the GTEx data set impede a comprehensive validation. Notably, the AAGMEx cohort was specifically designed for metabolism and aging studies, whereas the GTEx datasets originated from postmortem human tissues sourced primarily from European ancestry individuals. This difference may have influenced the level of gene expression (Ferreira et al., 2018). Consequently, while the GTEx cohort was larger, we observed less uniform and weaker aging associations compared to the AAGMEx cohort. This suggests that factors such as sample collection, sequencing, and analysis processes play a significant role. In our analysis of snRNA‐seq data for cell type‐specific gene regulations in adipose tissue, the donor size (*n* = 13) was relatively small for the pseudobulk analysis. To establish a more robust correlation, it is crucial to include more individuals from different aging stages. Additionally, while we used Spearman correlation to assess the strength and direction of monotonic relationships between variables, it is possible that complex nonlinear changes in aging regulations on metabolic rates and gene expressions may have been overlooked. Furthermore, the observed correlation between plasma metabolome data and transcriptome data from adipose and muscle tissues does not prove causality. Future work will require further validations, such as immunofluorescence and transgenic experiments, to establish the causal relationship and delve deeper into the dynamic aging‐associated expressions and functions of network hub genes in adipose and muscle tissues.

In summary, our study offers a comprehensive overview of cell‐type‐specific regulations and metabolic dysfunctions in glucose‐responsive tissues during aging. The findings emphasize the vulnerable cell types and aging‐associated regulatory networks in adipose and muscle tissues, which may contribute to the development of age‐related metabolic disorders. The connections between plasma metabolites and corresponding gene expressions in glucose‐responsive tissues suggest potential biomarkers and therapeutic targets for treating aging‐related disorders.

## 4 METHODS

### 4.1 The African American Genetics of Metabolism and Expression (AAGMEx) cohort

AAGMEx cohort consists of 256 unrelated, nondiabetic individuals who underwent multi‐omic profiling of plasma, adipose, and muscle tissues (Sajuthi et al., 2016; Sharma et al., 2016). For our analysis, we mainly focused on the transcriptome of 222 individuals with complete omics and phenotype data. The cohort consisted of healthy, self‐reported African Americans residing in North Carolina, including 123 men and 99 women, with an age range of 18–61 years and a BMI between 18 and 42 kg/m^2^. Abdominal subcutaneous adipose tissue near the umbilicus and *vastus lateralis* skeletal muscle biopsies were collected from participants after an overnight fast by Bergstrom needle under local anesthesia. The tissues were immediately rinsed in sterile saline, quick‐frozen in liquid nitrogen, and stored at −80°C. Fasting blood samples were also drawn for DNA isolation and biochemical analyses. All participants provided written informed consent under protocols approved by the institutional review boards at Wake Forest University School of Medicine.

### 4.2 Transcriptome profiling of adipose and muscle tissues

Transcriptome profiling in adipose and muscle tissues in the AAGMEx cohort was conducted according to previous publications (GEO ID #GSE95674 and #GSE95675 in super series #GSE95676) (Sajuthi et al., 2016; Sharma et al., 2016). Briefly, total RNA was extracted from adipose and muscle tissues using miRNeasy Mini Kit (Qiagen) and Ultraspec RNA total RNA extraction reagent (Biotecx laboratories), respectively. The extracted RNA was subjected to quality control using ultraviolet spectrophotometry (Nanodrop, Thermo Scientific) and electrophoresis (Experion nucleic acid analyzer, BioRad Laboratories, Inc.). Genome‐wide expression data were generated using HumanHT‐12 v4 Expression BeadChip (Illumina, San Diego, CA). The chips were scanned by the Illumina HiScan Reader using Illumina iScan Control Software, and probe‐level expressions were extracted using Illumina GenomeStudio V2011.1. The expression levels were log2 transformed, robust multi‐array average normalized (RMA, including quantile normalization), and batch‐corrected using ComBat. Transcripts that were not significantly expressed (*p*‐value < 0.05) in ≥25% of the samples and transcript probes encompassing common SNPs (based on ReAnnotator, or SnpInProbe annotation, and UCSC SNPv141) were excluded from downstream analyses.

### 4.3 DNA methylome profiling of adipose tissue

Epigenome‐wide profiling of DNA methylation in adipose was conducted and described in the previous study (Sharma et al., 2020). Genomic DNA was isolated from 100 mg frozen subcutaneous adipose biopsies using the Qiagen DNeasy tissue kit. Epigenome‐wide profiling of DNA methylation levels was conducted via reduced representation bisulfite sequencing (RRBS) by Diagenode RRBS service (Diagenode, Liege, Belgium). RRBS libraries were prepared and sequenced on a HiSeq3000 using 50 base pair single‐read sequencing to obtain at least 30 million reads/sample. The adapter‐trimmed sequence reads were aligned to the Homo sapiens reference genome (GRCh37, hg19) using Bismark v0.20.0 (Krueger & Andrews, 2011). MethylKit was utilized to filter and normalize the CpG data set (Akalin et al., 2012). We retained CpGs with at least 10X coverage, present in at least 75% of the samples, resulting in 1,073,614 CpGs. As previous study indicated that the average methylation level of a methylation region is likely to be robust to measurement errors (Orozco et al., 2018), methylation levels for 205,566 CpG regions were extracted using MethylKit, with 1000‐bp tiling windows across the genome, covering more than 10× in at least 75% of the samples. The R package “EnsDb.Hsapiens.v86” was used to extract 1 kb promoter regions of human genome and identify promoter‐located methylation of CpG regions.

### 4.4 Metabolome profiling of blood plasma

EDTA‐plasma samples were obtained from the AAGMEx cohort following overnight fasting and stored at −80°C until analysis. Untargeted liquid chromatography‐mass spectrometry was applied for metabolome profiling using the DiscoveryHD4 panel (Metabolon, Morrisville, NC). The resulting data was normalized by median‐scaling, log‐transformed, and missing values were imputed to the lowest measured value. Metabolites with missing data in more than 75% of the samples were excluded, leaving 1124 metabolites of good quality for downstream analyses.

### 4.5 RNA‐seq analysis of the GTEx data set

We obtained raw RNA‐seq data from human adipose and muscle tissues sourced from the GTEx (v8) database. For each tissue, we performed data normalization using the trimmed mean of M‐values (TMM) method to account for differences in sequencing library sizes (Robinson et al., 2010). The resulting normalized gene expression values were subjected to a log2 transformation. We also filtered out genes with low expression levels, defined as having log2 counts per million (CPM) value of ≤1 in over 75% of the total samples. We employed a linear model to examine correlations between gene expression and various traits (age, gender, BMI, and ethnicity), as well as covariates potentially contributing to batch effect and expression variation, including “SMCENTER” (collection sites), “SMRIN” (RNA integrity), “SMTSISCH” (ischemic time), “SMEXNCRT” (exonic rate), “SMRRNART” (rRNA rate), and “SMNTERRT” (intergenic rate). The beta coefficient and *p*‐value associated with age were extracted from the linear regression model and used for comparison analysis with the AAGMEx cohort.

### 4.6 MEGENA co‐expression network analysis

Before network construction, we normalized the transcripts from adipose and muscle tissues and metabolites from blood plasma using a multivariate regression model to remove the covariate effects of BMI and sex. We selected the transcript with the highest expression value for each gene for coexpression network analysis. The co‐expression network was constructed by the R package “MEGENA” (v1.4) following the recommended pipeline (Song & Zhang, 2015). Specifically, Pearson correlation coefficients (PCCs) were computed for all gene pairs to filter less correlated gene pairs by an FDR cutoff of 0.05. The ranked significant PCCs were iteratively tested for planarity to grow a Planar Filtered Network (PFN) using the Planar Maximally Filtered Graph (PMFG) algorithm. The resulting PFN was analyzed by Multiscale Clustering Analysis (MCA) to identify co‐expression modules at different network scale topologies. Multiscale Hub Analysis (MHA) was applied to identify module hub genes that are highly connected in each cluster.

### 4.7 Single‐cell RNA‐seq analysis

The single‐cell data sets of adipose and muscle tissues used in this study were obtained from publicly available sources (De Micheli et al., 2020; Emont et al., 2022). For adipose tissue, the normalized snRNA‐seq matrices and cell‐type annotations were obtained from the Single Cell Portal (Study #SCP1376), which includes 57,599 nuclei collected from human subcutaneous adipose tissue. For muscle tissue, the normalized scRNA‐seq matrices and cell‐type annotations were obtained from the GEO database under accession number GSE143704, which includes 22,000 single cells from a wide variety of anatomical sites. To identify marker genes in the two tissues of interest, we applied the R package Seurat (v3.9.9). Following previous studies, the “FindAllMarkers” function was used to identify differentially expressed genes using a Wilcoxon Rank Sum test (De Micheli et al., 2020; Emont et al., 2022). The marker gene lists were also verified using the MAST method, which is based on a two‐part hurdle model to handle bimodal zero‐distributed single‐cell data. Cell‐type marker genes were defined as significantly upregulated genes (FDR < 0.05) with 0.25 log2 fold change and 0.25 minimum expression fraction. In the pseudobulk analysis of the snRNA‐seq data set, gene counts within each cell type for each individual were aggregated. Subsequently, these counts were normalized using the trimmed mean of M‐values (TMM) method to correct for variations in sequencing library sizes (Robinson et al., 2010). The resultant normalized gene expression values were reported as counts per million (CPM) and underwent log2 transformation for subsequent analysis.

To estimate cell‐type fractions in bulk transcriptome data sets, we used BisqueRNA for deconvolution analysis, based on the snRNA‐seq gene count profile (Jew et al., 2020). The scRNA‐seq data sets from adipose and muscle tissues were obtained from previous studies (Emont et al., 2022; Perez et al., 2022). To identify the cell‐type specificity of network modules, we used Fisher's exact test (FET) from the R package clusterProfiler (v3.18). The enrichment *p* value was calculated by comparing the overlapping between the cell‐type marker genes and the module genes based on the deviation from a null hypothesis. We calculated the false discovery rate (FDR) using the Benjamini–Hochberg (BH) method to adjust for multiple testing.

### 4.8 Quantification and statistical analysis

To control for the effects of BMI and sex as covariates, a multivariate regression model was employed to adjust gene expressions, methylation levels in adipose and muscle tissues, and metabolite levels in blood plasma. Spearman correlation analysis, utilizing the adjusted values, was conducted to determine the relationship between age and these biological markers. Additionally, BMI values underwent square root transformation, while the S_I_ index was log‐transformed to perform correlation calculations. Enrichment tests were performed using the R package clusterProfiler (v3.18) for various purposes, including module pathway annotations and cell‐type enrichment analysis (Yu et al., 2012). The Benjamini–Hochberg (BH) FDR method was used to adjust for multi‐testing.

## AUTHOR CONTRIBUTIONS

PX and SKD perceived the concept and designed the study; PX performed the data analyses; PX, YK, and SKD wrote the paper; MCYN, NDP, and SKD contributed in metabolome data collection; and BZ supervised the network analysis. All the authors participated in the discussion and interpretation of the results, reviewed and revised the paper.

## CONFLICT OF INTEREST STATEMENT

The authors have no competing interests.

## Supporting information


Figures S1–S8.

Tables S1–S14.

## Data Availability

The summary of multi‐omic data sets reported in this study is available in Table S14. The full MEGENA networks and the metabolome data set can be downloaded through GitHub (https://github.com/penguab/AAGMEx_Aging_Network).

## References

[acel14199-bib-0001] Akalin, A. , Kormaksson, M. , Li, S. , Garrett‐Bakelman, F. E. , Figueroa, M. E. , Melnick, A. , & Mason, C. E. (2012). methylKit: A comprehensive R package for the analysis of genome‐wide DNA methylation profiles. Genome Biology, 13(10), R87. 10.1186/gb-2012-13-10-r87 23034086 PMC3491415

[acel14199-bib-0002] Akasaki, Y. , Ouchi, N. , Izumiya, Y. , Bernardo, B. L. , Lebrasseur, N. K. , & Walsh, K. (2014). Glycolytic fasttwitch muscle fiber restoration counters adverse age‐related changes in body composition and metabolism. Aging Cell, 13(1), 80–91. 10.1111/acel.12153 24033924 PMC3947044

[acel14199-bib-0003] Chai, J. C. , Chen, G. C. , Yu, B. , Xing, J. , Li, J. , Khambaty, T. , Perreira, K. M. , Perera, M. J. , Vidot, D. C. , Castaneda, S. F. , Selvin, E. , Rebholz, C. M. , Daviglus, M. L. , Cai, J. , Van Horn, L. , Isasi, C. R. , Sun, Q. , Hawkins, M. , Xue, X. , … Qi, Q. (2022). Serum metabolomics of incident diabetes and glycemic changes in a population with high diabetes burden: The Hispanic Community Health Study/Study of Latinos. Diabetes, 71(6), 1338–1349. 10.2337/db21-1056 35293992 PMC9163555

[acel14199-bib-0004] Chaleckis, R. , Murakami, I. , Takada, J. , Kondoh, H. , & Yanagida, M. (2016). Individual variability in human blood metabolites identifies age‐related differences. Proceedings of the National Academy of Sciences of the United States of America, 113(16), 4252–4259. 10.1073/pnas.1603023113 27036001 PMC4843419

[acel14199-bib-0005] Cooke, P. S. , Nanjappa, M. K. , Ko, C. , Prins, G. S. , & Hess, R. A. (2017). Estrogens in male physiology. Physiological Reviews, 97(3), 995–1043. 10.1152/physrev.00018.2016 28539434 PMC6151497

[acel14199-bib-0006] Das, S. S. , & Das, S. K. (2024). Common and ethnic‐specific derangements in skeletal muscle transcriptome associated with obesity. International Journal of Obesity, 48(3), 330–338. 10.1038/s41366-023-01417-y 37993634 PMC13310569

[acel14199-bib-0007] de Crecy‐Lagard, V. , Boccaletto, P. , Mangleburg, C. G. , Sharma, P. , Lowe, T. M. , Leidel, S. A. , & Bujnicki, J. M. (2019). Matching tRNA modifications in humans to their known and predicted enzymes. Nucleic Acids Research, 47(5), 2143–2159. 10.1093/nar/gkz011 30698754 PMC6412123

[acel14199-bib-0008] De Micheli, A. J. , Spector, J. A. , Elemento, O. , & Cosgrove, B. D. (2020). A reference single‐cell transcriptomic atlas of human skeletal muscle tissue reveals bifurcated muscle stem cell populations. Skeletal Muscle, 10(1), 19. 10.1186/s13395-020-00236-3 32624006 PMC7336639

[acel14199-bib-0009] DeFronzo, R. A. , & Tripathy, D. (2009). Skeletal muscle insulin resistance is the primary defect in type 2 diabetes. Diabetes Care, 32(Suppl 2), S157–S163. 10.2337/dc09-S302 19875544 PMC2811436

[acel14199-bib-0010] Dluzen, D. F. , Noren Hooten, N. , De, S. , Wood, W. H., 3rd , Zhang, Y. , Becker, K. G. , Zonderman, A. B. , Tanaka, T. , Ferrucci, L. , & Evans, M. K. (2018). Extracellular RNA profiles with human age. Aging Cell, 17(4), e12785. 10.1111/acel.12785 29797538 PMC6052399

[acel14199-bib-0011] Emont, M. P. , Jacobs, C. , Essene, A. L. , Pant, D. , Tenen, D. , Colleluori, G. , Di Vincenzo, A. , Jørgensen, A. M. , Dashti, H. , Stefek, A. , McGonagle, E. , Strobel, S. , Laber, S. , Agrawal, S. , Westcott, G. P. , Kar, A. , Veregge, M. L. , Gulko, A. , Srinivasan, H. , … Rosen, E. D. (2022). A single‐cell atlas of human and mouse white adipose tissue. Nature, 603, 926–933. 10.1038/s41586-022-04518-2 35296864 PMC9504827

[acel14199-bib-0012] Farup, J. , Just, J. , de Paoli, F. , Lin, L. , Jensen, J. B. , Billeskov, T. , Roman, I. S. , Cömert, C. , Møller, A. B. , Madaro, L. , Groppa, E. , Fred, R. G. , Kampmann, U. , Gormsen, L. C. , Pedersen, S. B. , Bross, P. , Stevnsner, T. , Eldrup, N. , Pers, T. H. , … Jessen, N. (2021). Human skeletal muscle CD90(+) fibro‐adipogenic progenitors are associated with muscle degeneration in type 2 diabetic patients. Cell Metabolism, 33(11), 2201–2214.e11. 10.1016/j.cmet.2021.10.001 34678202 PMC9165662

[acel14199-bib-0013] Ferreira, P. G. , Munoz‐Aguirre, M. , Reverter, F. , Sa Godinho, C. P. , Sousa, A. , Amadoz, A. , Sodaei, R. , Hidalgo, M. R. , Pervouchine, D. , Carbonell‐Caballero, J. , Nurtdinov, R. , Breschi, A. , Amador, R. , Oliveira, P. , Çubuk, C. , Curado, J. , Aguet, F. , Oliveira, C. , Dopazo, J. , … Guigo, R. (2018). The effects of death and post‐mortem cold ischemia on human tissue transcriptomes. Nature Communications, 9(1), 490. 10.1038/s41467-017-02772-x PMC5811508 29440659

[acel14199-bib-0014] Heine, P. A. , Taylor, J. A. , Iwamoto, G. A. , Lubahn, D. B. , & Cooke, P. S. (2000). Increased adipose tissue in male and female estrogen receptor‐alpha knockout mice. Proceedings of the National Academy of Sciences of the United States of America, 97(23), 12729–12734. 10.1073/pnas.97.23.12729 11070086 PMC18832

[acel14199-bib-0015] Jew, B. , Alvarez, M. , Rahmani, E. , Miao, Z. , Ko, A. , Garske, K. M. , Sul, J. H. , Pietiläinen, K. H. , Pajukanta, P. , & Halperin, E. (2020). Accurate estimation of cell composition in bulk expression through robust integration of single‐cell information. Nature Communications, 11(1), 1971. 10.1038/s41467-020-15816-6 PMC7181686 32332754

[acel14199-bib-0016] Jones, M. J. , Goodman, S. J. , & Kobor, M. S. (2015). DNA methylation and healthy human aging. Aging Cell, 14(6), 924–932. 10.1111/acel.12349 25913071 PMC4693469

[acel14199-bib-0017] Kedlian, V. R. , Wang, Y. , Liu, T. , Chen, X. , Bolt, L. , Tudor, C. , Shen, Z. , Fasouli, E. S. , Prigmore, E. , Kleshchevnikov, V. , Pett, J. P. , Li, T. , Lawrence, J. E. G. , Perera, S. , Prete, M. , Huang, N. , Guo, Q. , Zeng, X. , Yang, L. , … Zhang, H. (2024). Human skeletal muscle aging atlas. Nature Aging. 10.1038/s43587-024-00613-3 PMC11108788 38622407

[acel14199-bib-0018] Kershaw, E. E. , & Flier, J. S. (2004). Adipose tissue as an endocrine organ. The Journal of Clinical Endocrinology and Metabolism, 89(6), 2548–2556. 10.1210/jc.2004-0395 15181022

[acel14199-bib-0019] Kirkland, J. L. , Tchkonia, T. , Pirtskhalava, T. , Han, J. , & Karagiannides, I. (2002). Adipogenesis and aging: does aging make fat go MAD? Experimental Gerontology, 37(6), 757–767. 10.1016/s0531-5565(02)00014-1 12175476

[acel14199-bib-0020] Krueger, F. , & Andrews, S. R. (2011). Bismark: A flexible aligner and methylation caller for bisulfite‐seq applications. Bioinformatics, 27(11), 1571–1572. 10.1093/bioinformatics/btr167 21493656 PMC3102221

[acel14199-bib-0021] Lopez‐Otin, C. , Blasco, M. A. , Partridge, L. , Serrano, M. , & Kroemer, G. (2023). Hallmarks of aging: An expanding universe. Cell, 186(2), 243–278. 10.1016/j.cell.2022.11.001 36599349

[acel14199-bib-0022] Orozco, L. D. , Farrell, C. , Hale, C. , Rubbi, L. , Rinaldi, A. , Civelek, M. , Pan, C. , Lam, L. , Montoya, D. , Edillor, C. , Seldin, M. , Boehnke, M. , Mohlke, K. L. , Jacobsen, S. , Kuusisto, J. , Laakso, M. , Lusis, A. J. , & Pellegrini, M. (2018). Epigenomewide association in adipose tissue from the METSIM cohort. Human Molecular Genetics, 27(10), 1830–1846. 10.1093/hmg/ddy093 29566149 PMC5932563

[acel14199-bib-0023] Ou, M. Y. , Zhang, H. , Tan, P. C. , Zhou, S. B. , & Li, Q. F. (2022). Adipose tissue aging: Mechanisms and therapeutic implications. Cell Death & Disease, 13(4), 300. 10.1038/s41419-022-04752-6 35379822 PMC8980023

[acel14199-bib-0024] Parra‐Peralbo, E. , Talamillo, A. , & Barrio, R. (2021). Origin and development of the adipose tissue, a key organ in physiology and disease. Frontiers in Cell and Development Biology, 9, 786129. 10.3389/fcell.2021.786129 PMC8724577 34993199

[acel14199-bib-0025] Pataky, M. W. , Young, W. F. , & Nair, K. S. (2021). Hormonal and metabolic changes of aging and the influence of lifestyle modifications. Mayo Clinic Proceedings, 96(3), 788–814. 10.1016/j.mayocp.2020.07.033 33673927 PMC8020896

[acel14199-bib-0026] Perez, K. , Ciotlos, S. , McGirr, J. , Limbad, C. , Doi, R. , Nederveen, J. P. , Nilsson, M. I. , Winer, D. A. , Evans, W. , Tarnopolsky, M. , Campisi, J. , & Melov, S. (2022). Single nuclei profiling identifies cell specific markers of skeletal muscle aging, frailty, and senescence. Aging (Albany NY), 14(23), 9393–9422. 10.18632/aging.204435 36516485 PMC9792217

[acel14199-bib-0027] Periasamy, M. , & Kalyanasundaram, A. (2007). SERCA pump isoforms: Their role in calcium transport and disease. Muscle & Nerve, 35(4), 430–442. 10.1002/mus.20745 17286271

[acel14199-bib-0028] Richardson, N. E. , Konon, E. N. , Schuster, H. S. , Mitchell, A. T. , Boyle, C. , Rodgers, A. C. , Finke, M. , Haider, L. R. , Yu, D. , Flores, V. , Pak, H. H. , Ahmad, S. , Ahmed, S. , Radcliff, A. , Wu, J. , Williams, E. M. , Abdi, L. , Sherman, D. S. , Hacker, T. , & Lamming, D. W. (2021). Lifelong restriction of dietary branched‐chain amino acids has sex‐specific benefits for frailty and lifespan in mice. Nature Aging, 1(1), 73–86. 10.1038/s43587-020-00006-2 33796866 PMC8009080

[acel14199-bib-0029] Robinson, M. D. , McCarthy, D. J. , & Smyth, G. K. (2010). edgeR: A Bioconductor package for differential expression analysis of digital gene expression data. Bioinformatics, 26(1), 139–140. 10.1093/bioinformatics/btp616 19910308 PMC2796818

[acel14199-bib-0030] Saito, K. , Maekawa, K. , Kinchen, J. M. , Tanaka, R. , Kumagai, Y. , & Saito, Y. (2016). Gender‐ and ageassociated differences in serum metabolite profiles among Japanese populations. Biological and Pharmaceutical Bulletin, 39(7), 1179–1186. 10.1248/bpb.b16-00226 27374292

[acel14199-bib-0031] Sajuthi, S. P. , Sharma, N. K. , Chou, J. W. , Palmer, N. D. , McWilliams, D. R. , Beal, J. , Comeau, M. E. , Ma, L. , Calles‐Escandon, J. , Demons, J. , Rogers, S. , Cherry, K. , Menon, L. , Kouba, E. , Davis, D. , Burris, M. , Byerly, S. J. , Ng, M. C. , Maruthur, N. M. , … Das, S. K. (2016). Mapping adipose and muscle tissue expression quantitative trait loci in African Americans to identify genes for type 2 diabetes and obesity. Human Genetics, 135(8), 869–880. 10.1007/s00439-016-1680-8 27193597 PMC4947558

[acel14199-bib-0032] Schiaffino, S. , & Reggiani, C. (2011). Fiber types in mammalian skeletal muscles. Physiological Reviews, 91(4), 14471531. 10.1152/physrev.00031.2010 22013216

[acel14199-bib-0033] Schöneberg, T. , Kloos, M. , Brüser, A. , Kirchberger, J. , & Sträter, N. (2013). Structure and allosteric regulation of eukaryotic 6‐phosphofructokinases. Biological Chemistry, 394(8), 977–993. 10.1515/hsz-2013-0130 23729568

[acel14199-bib-0034] Seldin, M. M. , Koplev, S. , Rajbhandari, P. , Vergnes, L. , Rosenberg, G. M. , Meng, Y. , Pan, C. , Phuong, T. M. N. , Gharakhanian, R. , Che, N. , Mäkinen, S. , Shih, D. M. , Civelek, M. , Parks, B. W. , Kim, E. D. , Norheim, F. , Chella Krishnan, K. , Hasin‐Brumshtein, Y. , Mehrabian, M. , … Lusis, A. J. (2018). A strategy for discovery of endocrine interactions with application to whole‐body metabolism. Cell Metabolism, 27(5), 1138–1155 e1136. 10.1016/j.cmet.2018.03.015 29719227 PMC5935137

[acel14199-bib-0035] Sharma, N. K. , Comeau, M. E. , Montoya, D. , Pellegrini, M. , Howard, T. D. , Langefeld, C. D. , & Das, S. K. (2020). Integrative analysis of glucometabolic traits, adipose tissue DNA methylation, and gene expression identifies epigenetic regulatory mechanisms of insulin resistance and obesity in African Americans. Diabetes, 69(12), 2779–2793. 10.2337/db20-0117 32928872 PMC7679782

[acel14199-bib-0036] Sharma, N. K. , Sajuthi, S. P. , Chou, J. W. , Calles‐Escandon, J. , Demons, J. , Rogers, S. , Ma, L. , Palmer, N. D. , McWilliams, D. R. , Beal, J. , Comeau, M. E. , Cherry, K. , Hawkins, G. A. , Menon, L. , Kouba, E. , Davis, D. , Burris, M. , Byerly, S. J. , Easter, L. , … Das, S. K. (2016). Tissue‐specific and genetic regulation of insulin sensitivity‐associated transcripts in African Americans. The Journal of Clinical Endocrinology and Metabolism, 101(4), 1455–1468. 10.1210/jc.2015-3336 26789776 PMC4880154

[acel14199-bib-0037] Song, W. M. , & Zhang, B. (2015). Multiscale embedded gene co‐expression network analysis. PLoS Computational Biology, 11(11), e1004574. 10.1371/journal.pcbi.1004574 26618778 PMC4664553

[acel14199-bib-0038] Sousa‐Victor, P. , Garcia‐Prat, L. , Serrano, A. L. , Perdiguero, E. , & Munoz‐Canoves, P. (2015). Muscle stem cell aging: Regulation and rejuvenation. Trends in Endocrinology and Metabolism, 26(6), 287–296. 10.1016/j.tem.2015.03.006 25869211

[acel14199-bib-0039] Van Hove, J. L. , Saenz, M. S. , Thomas, J. A. , Gallagher, R. C. , Lovell, M. A. , Fenton, L. Z. , Shanske, S. , Myers, S. M. , Wanders, R. J. , Ruiter, J. , Turkenburg, M. , & Waterham, H. R. (2010). Succinyl‐CoA ligase deficiency: A mitochondrial hepatoencephalomyopathy. Pediatric Research, 68(2), 159–164. 10.1203/PDR.0b013e3181e5c3a4 20453710 PMC2928220

[acel14199-bib-0040] Wilkinson, D. J. , Piasecki, M. , & Atherton, P. J. (2018). The age‐related loss of skeletal muscle mass and function: Measurement and physiology of muscle fibre atrophy and muscle fibre loss in humans. Ageing Research Reviews, 47, 123–132. 10.1016/j.arr.2018.07.005 30048806 PMC6202460

[acel14199-bib-0041] Wolfson, R. L. , Chantranupong, L. , Saxton, R. A. , Shen, K. , Scaria, S. M. , Cantor, J. R. , & Sabatini, D. M. (2016). Sestrin2 is a leucine sensor for the mTORC1 pathway. Science, 351(6268), 43–48. 10.1126/science.aab2674 26449471 PMC4698017

[acel14199-bib-0042] Wu, D. , Ren, Z. , Pae, M. , Guo, W. , Cui, X. , Merrill, A. H. , & Meydani, S. N. (2007). Aging up‐regulates expression of inflammatory mediators in mouse adipose tissue. Journal of Immunology, 179(7), 4829–4839. 10.4049/jimmunol.179.7.4829 17878382

[acel14199-bib-0043] Xu, P. , Wang, M. , Sharma, N. K. , Comeau, M. E. , Wabitsch, M. , Langefeld, C. D. , Civelek, M. , Zhang, B. , & Das, S. K. (2023). Multi‐omic integration reveals cell‐type‐specific regulatory networks of insulin resistance in distinct ancestry populations. Cell Systems, 14(1), 41–57.e48. 10.1016/j.cels.2022.12.005 36630956 PMC9852073

[acel14199-bib-0044] Xu, P. , Wang, M. , Song, W. M. , Wang, Q. , Yuan, G. C. , Sudmant, P. H. , Zare, H. , Tu, Z. , Orr, M. E. , & Zhang, B. (2022). The landscape of human tissue and cell type specific expression and co‐regulation of senescence genes. Molecular Neurodegeneration, 17(1), 5. 10.1186/s13024-021-00507-7 35000600 PMC8744330

[acel14199-bib-0045] Yu, G. , Wang, L. G. , Han, Y. , & He, Q. Y. (2012). clusterProfiler: An R package for comparing biological themes among gene clusters. OMICS, 16(5), 284–287. 10.1089/omi.2011.0118 22455463 PMC3339379

