# Supplementary material for: Integrated multi‐omic analyses uncover the effects of aging on cell‐type regulation in glucose‐responsive tissues
Source: Aging Cell. 2024 Jun 26;23(8):e14199. doi: 10.1111/acel.14199 (PMC11320340; doi:10.1111/acel.14199)
Supplement: Supplementary file 1 — Figures S1–S8. [file ACEL-23-e14199-s002.docx]

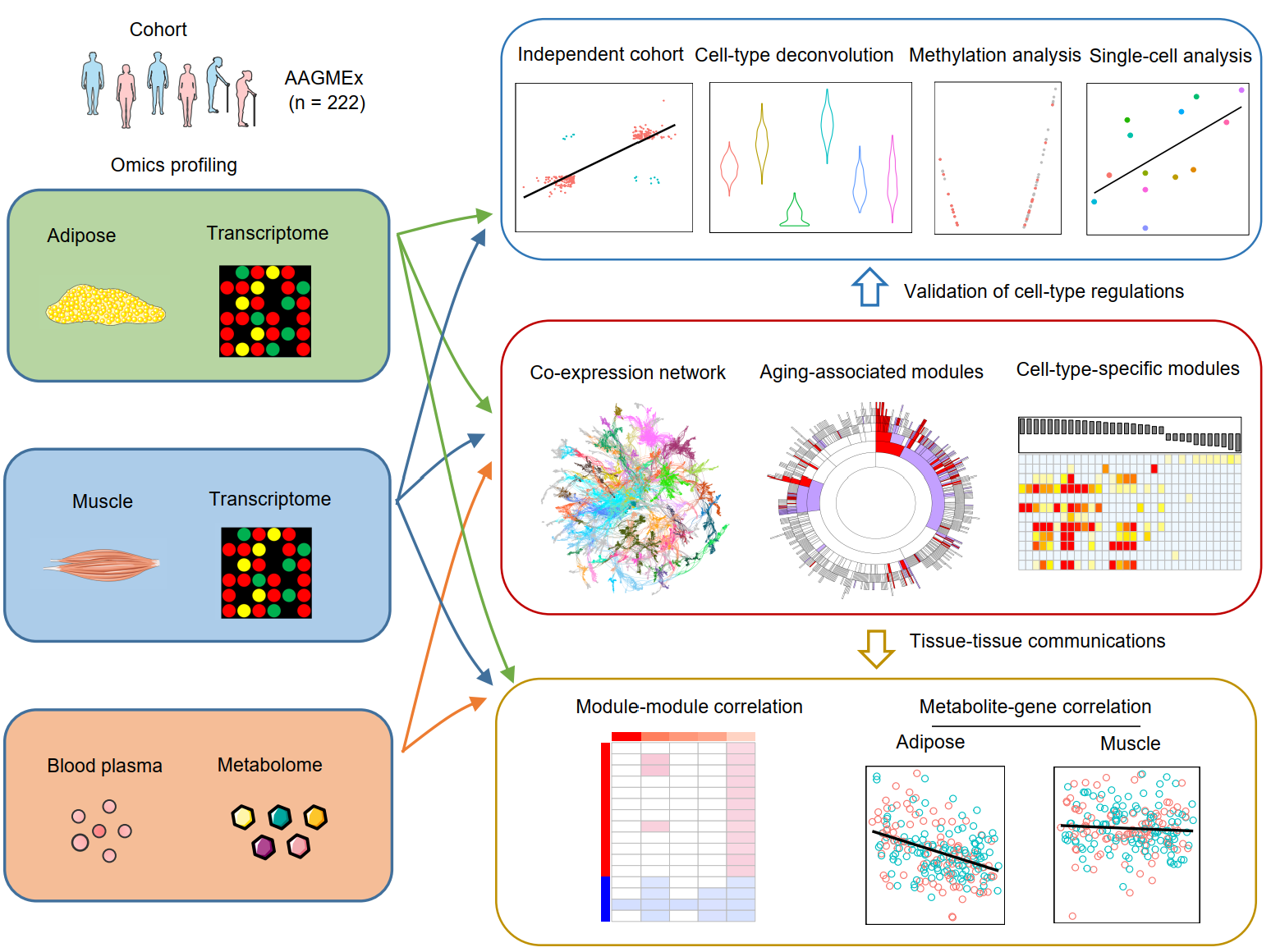


**Fig. S1 Diagram of research goal, study design, and data analysis workflow.**


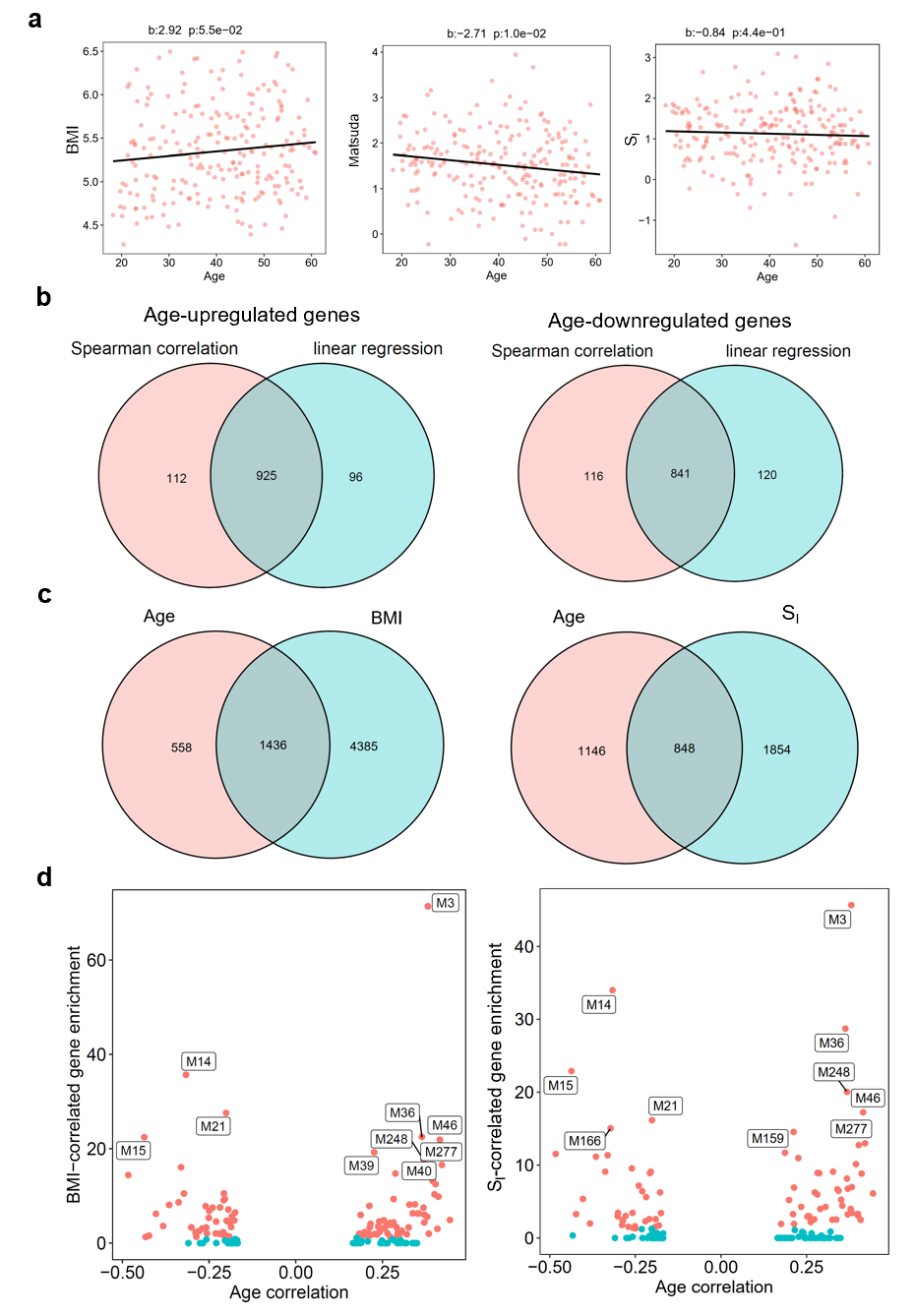


**Fig. S2 Influence of age, BMI, and S_I_ on gene expressions in adipose tissue.** **a,** Linear regression analysis between age and other traits, including BMI and insulin sensitivity index (Matsuda and S_I_), in the AAGMEx cohort. The beta coefficient and p-value of the regression model are labeled in the plot title. **b,** Venn diagrams showing the overlap between age-regulated genes identified by Spearman correlation and linear regression analyses (FDR < 0.05). **c**, Venn diagrams showing genes co-regulated by age and the other two factors (BMI and S_I_) in adipose tissue. **d,** Enrichment of BMI- and S_I_-correlated genes in the age-associated modules of adipose tissue. The x-axis indicates the correlation coefficients between module eigengenes and age, and the y-axis indicates the enrichment significance.


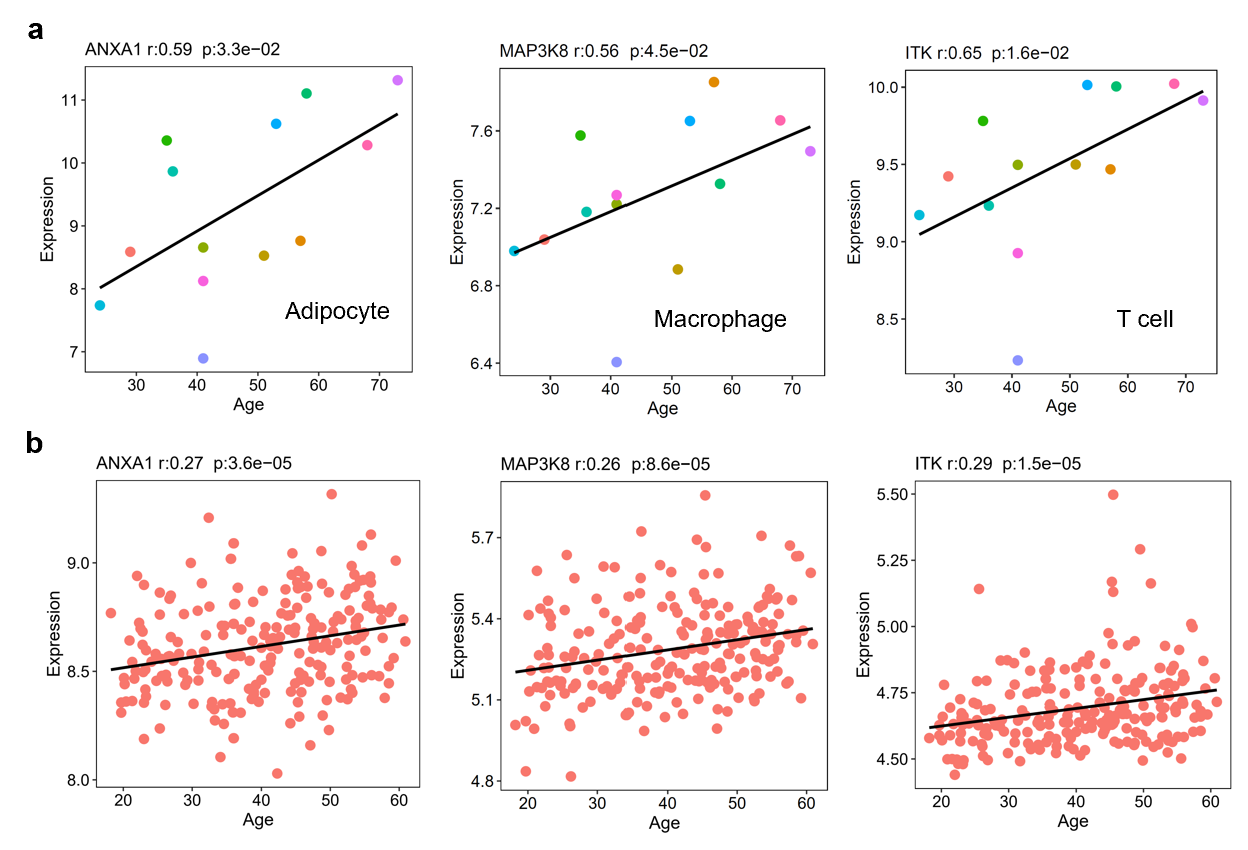


**Fig. S3 Cell-type-specific gene expression changes associated with aging in adipose tissue. a,** Pseudobulk analysis of representative regulators in adipose tissue with aging. Each dot represents gene expression in a particular cell type of an individual. The Spearman correlation coefficient and p-value for the age association are annotated in the plot title. **b,** Point plots showing aging association of representative adipose regulators in AAGMEx bulk sequencing dataset.


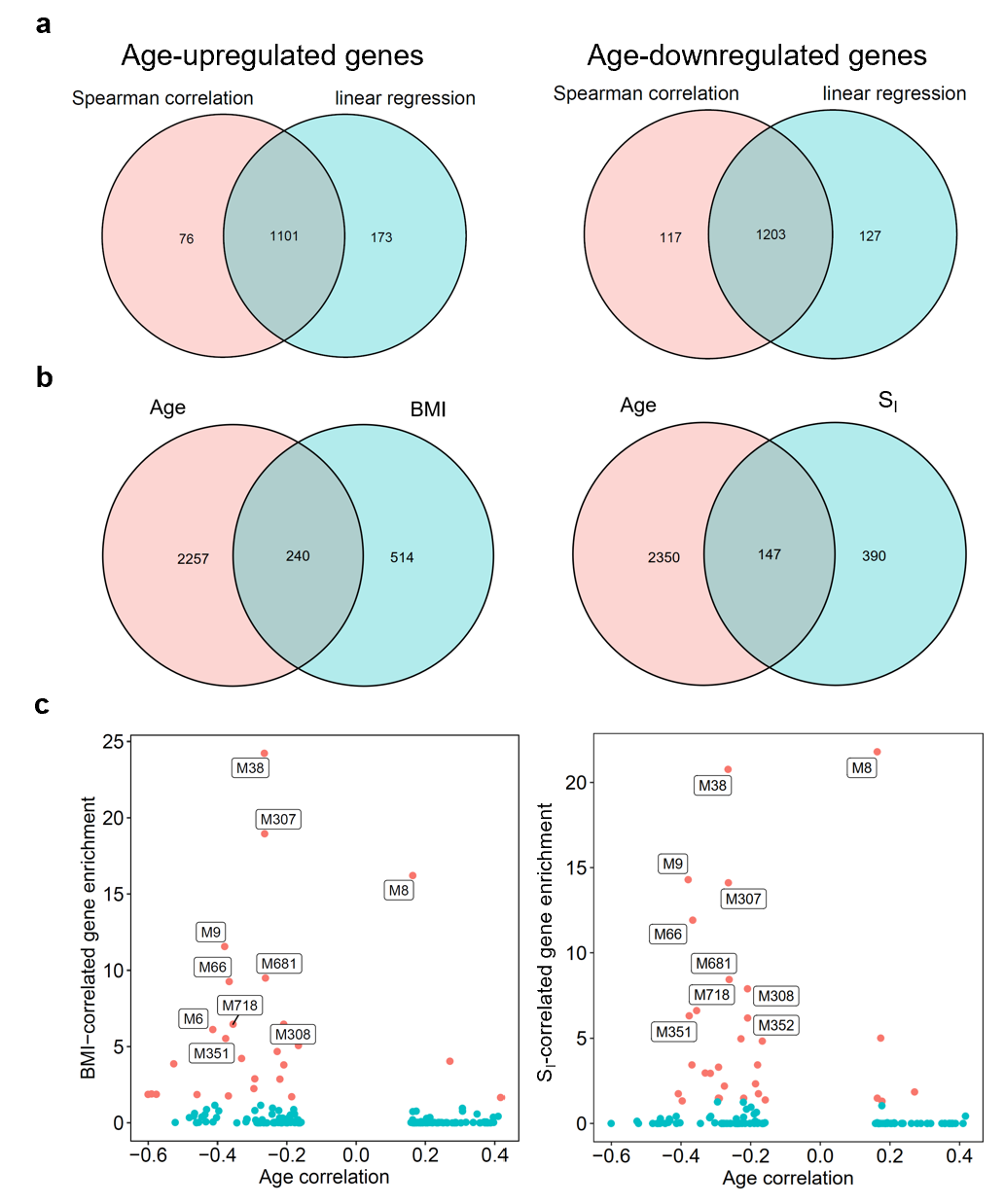


**Fig. S4 Influence of age, BMI, and S_I_ on gene expressions in muscle tissue. a,** Venn diagrams showing the overlap between age-regulated genes identified by Spearman correlation and linear regression analyses in the AAGMEx cohort (FDR < 0.05). **b,** Venn diagrams showing genes co-regulated by age and the other two factors (BMI and S_I_) in muscle tissue. A multivariate linear regression model was applied to calculate the influence of age and other factors on gene expressions. **c,** Enrichment of BMI- and S_I_-correlated genes in the age-associated modules of muscle tissue. The x-axis indicates the correlation coefficients between module eigengenes and age, and the y-axis indicates the enrichment significance.


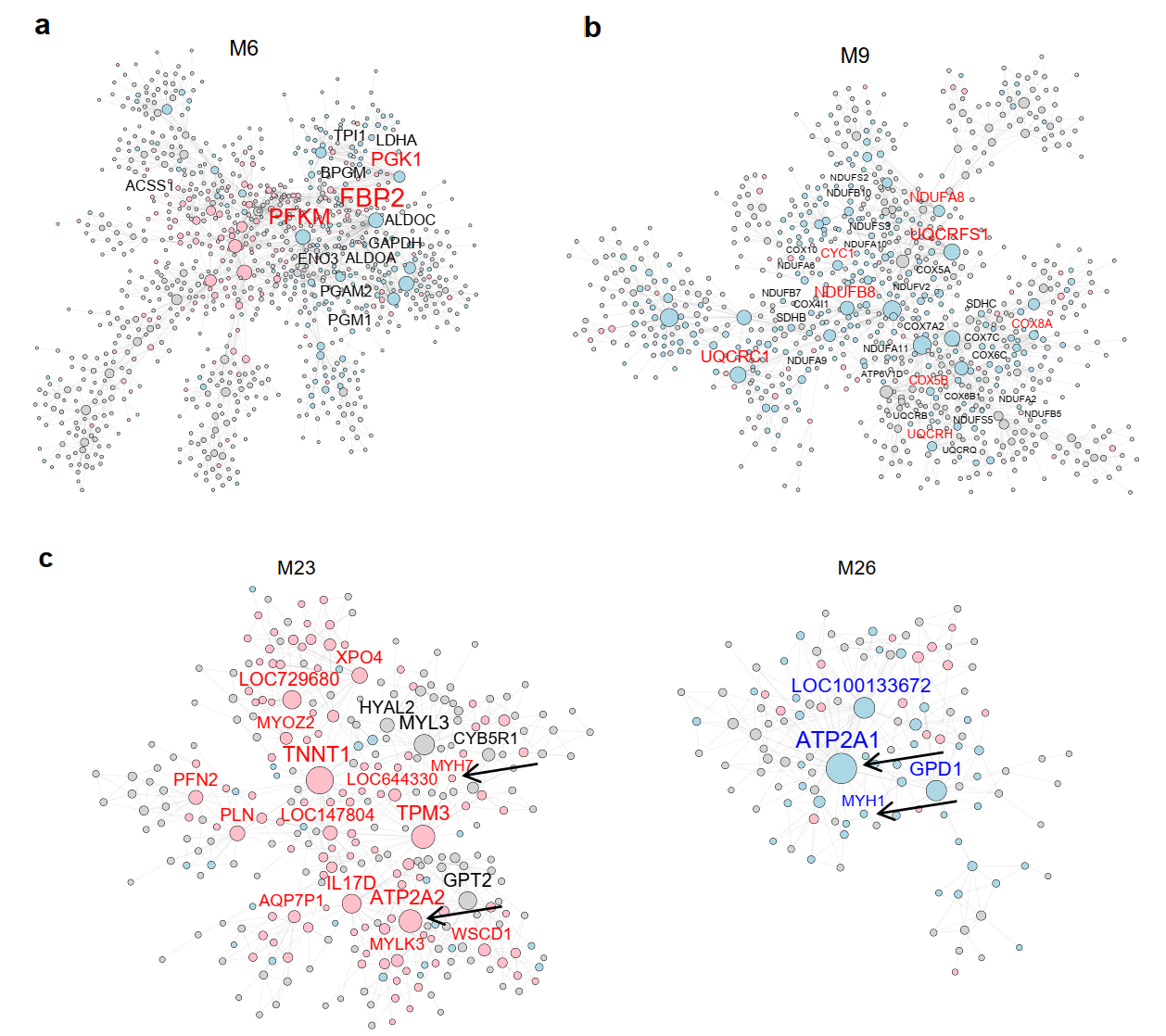


**Fig. S5 The aging-correlated network modules in different muscle fiber types. a-b**, Network visualization of network modules enriched for the glycolysis pathway (M6, **a**) and the oxidative phosphorylation pathway (M9, **b**). Nodes represent genes, and links indicate co-expression relationships. Dot size correlates with network connectivity, and color (pink/blue) indicates positive/negative age correlations. The names of age-associated genes from each pathway are labeled above the nodes, with red color indicating network hub genes. **c,** Network modules specific to different muscle fiber types. Modules M23 and M26 contain marker genes specific to Type Ⅰ and Ⅱ muscle fibers, respectively, with opposing aging regulations. Fiber-type marker genes are marked with black arrows.


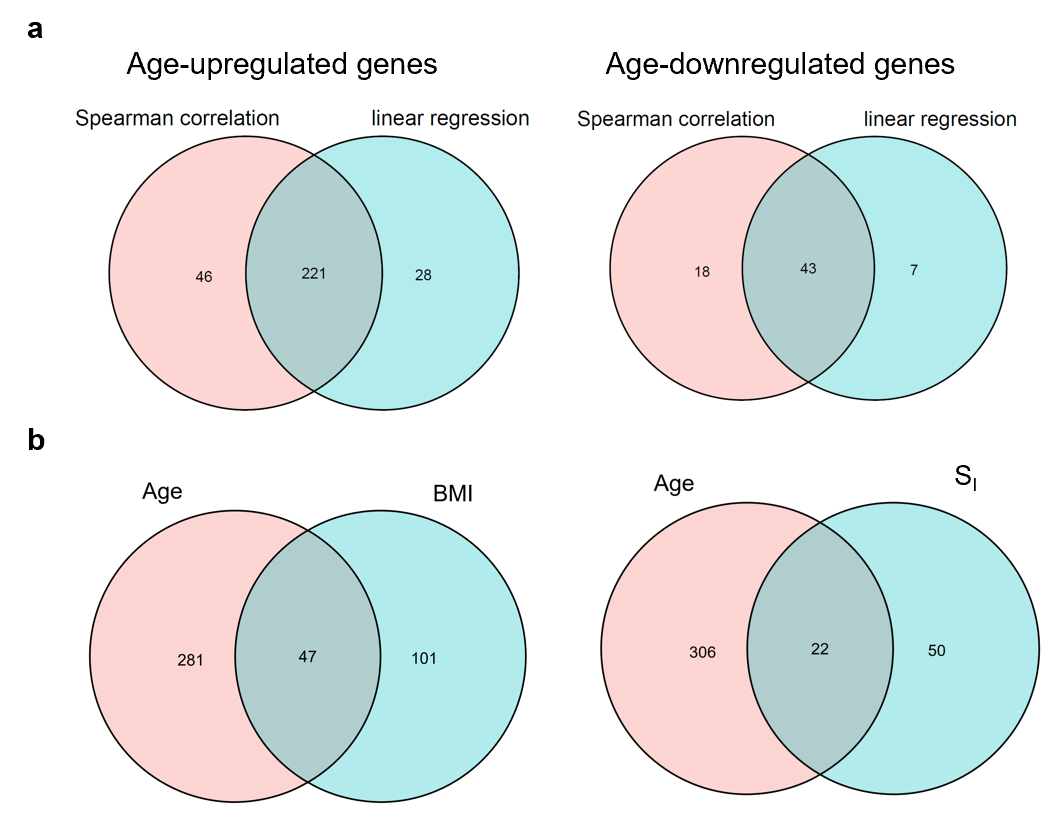


**Fig. S6 Influence of age, BMI, and S_I_ on metabolite levels in blood plasma. a,** Venn diagrams showing the overlap between age-regulated metabolites identified by Spearman correlation and linear regression analyses in the AAGMEx cohort (FDR < 0.05). **b,** Venn diagrams showing metabolites co-regulated by age and the other two factors (BMI and S_I_) in blood plasma. A multivariate linear regression model was applied to calculate the influence of age and other factors on metabolite expressions.


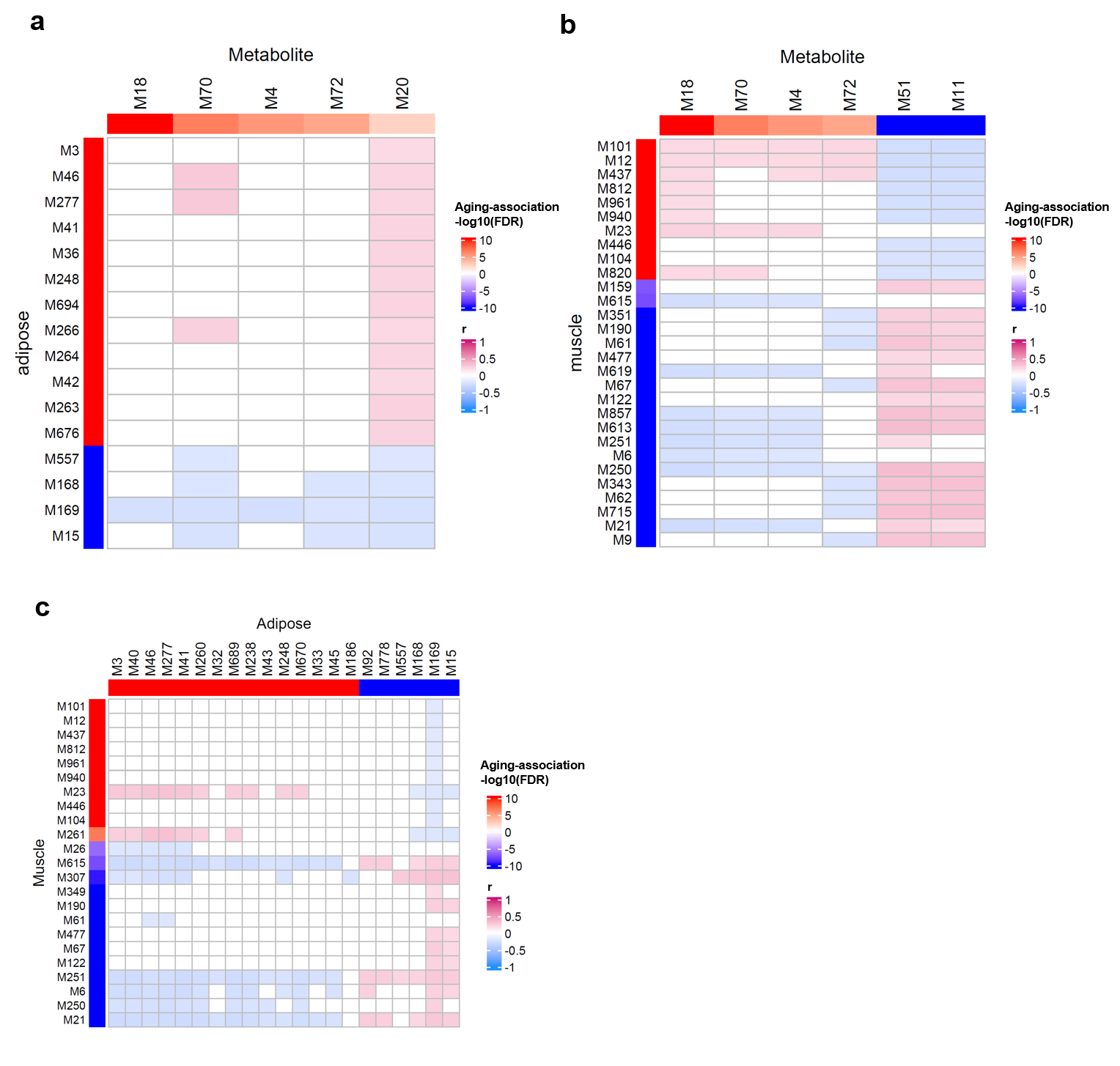


**Fig. S7 Correlation of aging-associated network modules between plasma metabolome and transcriptome of glucose-responsive tissues. a-b,** The heatmap plots displaying the Spearman correlation coefficients of aging-associated modules between plasma metabolites and adipose **(a)** and muscle **(b)** tissues. The color bar on the top and left sides of the heatmap plot shows the module enrichment (-log10(FDR), FET) of differentially expressed metabolites and genes during aging, with the negative values indicating down-regulation. The top 30 aging-associated modules from each tissue were selected for pair-wise correlation analysis. **c,** The heatmap plot displaying the Spearman correlation coefficients of aging-associated modules between adipose and muscle tissues.


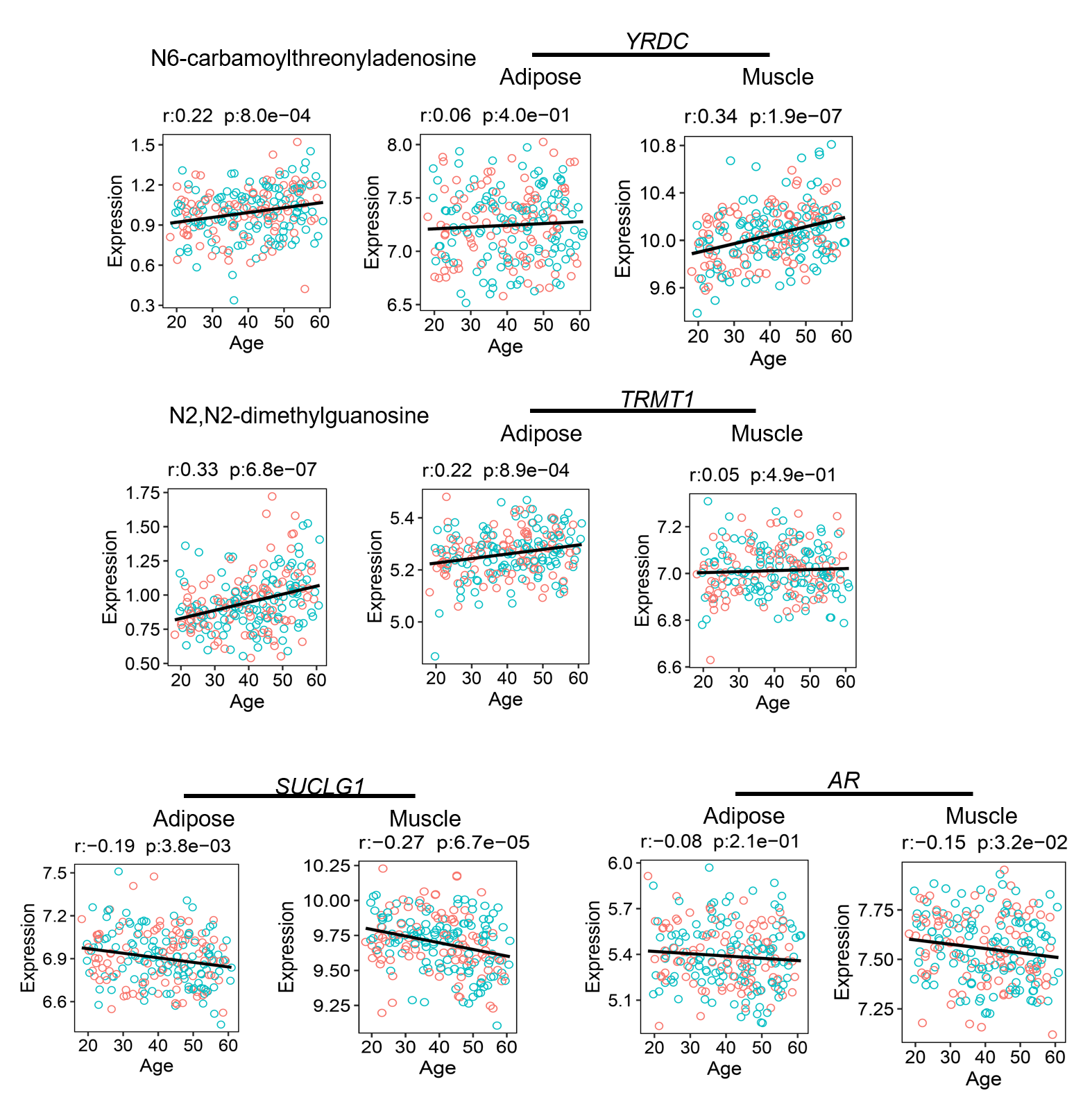


**Fig. S8 Examples of age-associated metabolites and their biosynthesis or pathway genes in adipose and muscle tissues.** The dot plots display the correlations between age and the expressions of metabolites or genes. The red and blue colors represent data from female and male samples, respectively. The black line represents the slope of a linear regression model. The Spearman correlation coefficient and p-value are labeled in the plot title.
